# Quantitative proteomic analysis of local and systemic extracellular vesicles during *Eimeria falciformis* infectious cycle in the host

**DOI:** 10.1186/s13071-023-05906-x

**Published:** 2023-09-27

**Authors:** Joshua Seun Olajide, Zigang Qu, Shunli Yang, Bin Yang, Xiao Xu, Jing Wang, Jianping Cai

**Affiliations:** 1 State Key Laboratory of Animal Disease Control and Prevention, Key Laboratory of Veterinary Parasitology of Gansu, Lanzhou Veterinary Research Institute, Chinese Academy of Agricultural Sciences, Lanzhou, 730046 China; 2 Centre for Distance Learning, Obafemi Awolowo University, Ile-Ife, Nigeria; 3 College of Animal Science and Technology, Hebei Normal University of Science and Technology, Qinhuangdao, China

**Keywords:** Extracellular vesicles, Proteins, *Eimeria falciformis*, Tandem mass tag, Coccidiosis

## Abstract

**Background:**

Extracellular vesicles (EVs) are membranous structures that are formed during pathophysiology, host-parasite interactions and parasite motility. Typically, apicomplexan-infected host cells secrete EVs which traverse local and systemic strata of the host as the parasites develop.

**Methods:**

Extracellular vesicles were isolated from the caecum and serum of *Eimeria falciformis*-infected mice during oocyst ingestion (0 h post-infection [0 hpi]), merozont stages 1 and 2 (68 and 116 hpi), oocyst shedding (7 days post-infection [7 dpi]) and host recovery (10 dpi) and subsequently characterized and profiled by tandem mass tag (TMT).

**Results:**

With the progression of *E. falciformis* life stages, subpopulation of EVs bearing EV biomarkers, including CD9, CD82, heat shock protein 70 (HSP70) and major histocompatibility complex (MHC) molecules, increased. A total of 860 and 1024 differentially expressed proteins were identified in serum EVs (sEVs) and caecum EVs (cEVs), respectively. Identified immune-related molecules (such as cytokines, receptors, immunoglobins, complements, hormones, inflammasomes), ion exchange and cell death-associated proteins were significantly expressed, at least during the *E. falciformis* first and second merozont stages. Bioinformatics assessment indicated that sEV proteins were at all time points implicated in antigen processing and presentation as well as natural killer cell-mediated cytotoxicity (68 hpi), complement activation/blood coagulation (116 hpi/10 dpi) and catabolic activities (7 dpi). In contrast, cEV proteins were involved in catabolic process, ion transport and antigen presentation (68 and 116 hpi). Host response to *E. falciformis* infection was similar to intestinal bacterium at 7 dpi and cell adhesion and intercellular protein transport at 10 dpi. In both systems, ferroptosis and necroptosis were common across the parasite’s infectious cycle while apoptosis occurred at 68 hpi.

**Conclusion:**

The proteomic data indicate that *E. falciformis* infection co-opts cellular and humoral responses through EV secretions, and that, host cell death and ionic imbalance are associated with *E. falciformis* infection. This study offers additional insight into host-parasite interactions and host regulatory EV proteins as potential disease indicators or diagnostic molecules.

**Graphical Abstract:**

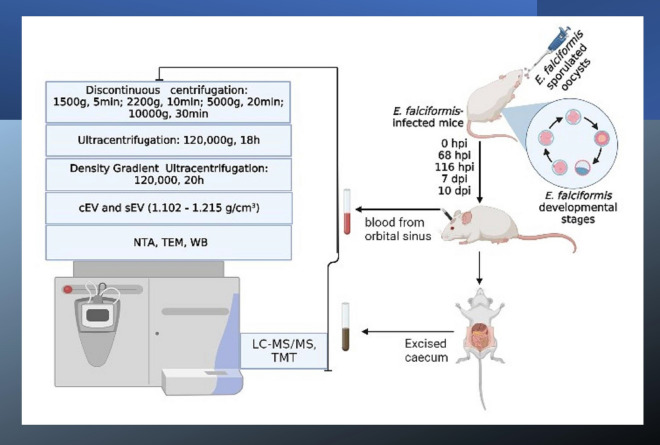

**Supplementary Information:**

The online version contains supplementary material available at 10.1186/s13071-023-05906-x.

## Background

Extracellular vesicles (EVs) are submicron membrane-bound structures secreted by all cell types during cellular stress, diseases and pathophysiology [1, 2]. EVs are generally heterogeneous in size, ranging from 30 nm to 4 µm [3], and broadly classified as exosomes, ectosomes (microparticles or microvesicles) and apoptotic bodies [4, 5]. These structures are important disease biomarkers (either of the host or the parasite), pathological indicators and therapeutic tools [6]. Signal transduction, cytokines and immune peptides [2, 7], transmembrane proteins, growth factors, bioactive lipids, carbohydrates, nucleic acids and episomal genes [4, 8] are among the various molecules usually encapsulated in EVs.

The secretion of EVs is also central to parasite-host interactions and pathogenesis [9], host response to parasite invasion [10], parasite-induced stressors [11, 12], parasite-parasite communications and pathogenesis [12]. As well, EVs are important in parasite developmental transitions and maturation [14, 15]. EVs are also deployed to modulate disruption and cytolysis of parasite-uninfected host cells, and as antigen presenters and danger signals to immune cells [8, 13]. During parasite infection, EVs are able to transverse local, systemic and lymphatic circulation [14] and be carried to distantly located organs in the host [4, 10].

Members of prominent Apicomplexan genera, including *Plasmodium*, *Toxoplasma*, *Cryptosporidium, Theileria* and *Eimeria*, are important human and animal parasites [15]. *Eimeria falciformis* is considered to be the most prevalent parasite of laboratory and wild mice [16], and a model species to study *Eimeria*-host interaction [15]. *Eimeria. falcifromis* parasitizes the host’s caecum and has a monoxenous life-cycle [20, 21]. After ingestion of *E. falciformis* sporulated oocysts by the host, the parasite undergoes excystment to release sporocysts first, followed by sporozoites that migrate to the caecum and invade intestinal epithelial cells (IECs). This is followed by four generations of merozonts after which micro- and macro-gametes are formed and fused to form zygotes which later develop as oocysts shed in host faeces [18, 20]. The entire *E. falciformis* life-cycle occurs in about 13 days [19]. Pathologically, *E. falciformis* infection is characterized by catarrhal enteritis, haemorrhage, epithelial sloughing, diarrhea, inflammation, infiltration of immune cell and host death [24, 25].

As *E. falciformis* development continues in the host, diverse overlapping secretory events occur. Also, pathogenesis by the coccidian parasite could extend to distant organs through EVs that migrate from the site of parasite infection to distant tissues via the circulatory system [4]. Although *E. falciformis* developmental stages have been described [17, 22, 24], this study was designed to elucidate the composition and regulation of proteins in *E. falciformis*-infected mice caecum-derived EVs (cEVs) and serum-derived EVs (sEVs) during the parasite infectious cycle in the host.

## Methods

### Ethical statement

Mice were handled in strict compliance with Animal Ethical Procedures and Guidelines of the People’s Republic of China as approved by Animal Administration and Ethics Committee of Lanzhou Veterinary Research Institute, Chinese Academy of Agricultural Sciences (LVRIAEC-2020-035).

### Experimental infection and sample collection

Kunming mice of the Swiss-line strain [26], aged between 6 and 8 eight weeks and with a mean weight of 23 ± 1.68 g, were confirmed parasite-free by microscopic observation of faecal smears and indirect enzyme-linked immunosorbent assay (ELISA) using antibodies developed against *E. falciformis* sporulated oocysts [22]. The mice were then orally inoculated with 50 µl of PBS containing 2 × 10^5^ *E. falciformis* sporulated oocysts per millilitre of PBS. Parasite-infected mice were randomly chosen and euthanized at 0, 68, 116 h post-infection (hpi) and 7 and 10 days post-infection (dpi). Blood was collected from the euthanized mice through the orbital sinus into sterilized bottles [27, 28]. Prior to collecting caecal tissues, the ventral side of each mouse was sprayed with 70% ethanol and wiped with gauze pads [24]. The abdominal cavity was then cut open, and tissue approximately 2 cm equidistance from the caecum was excised. The tissues were immediately snap-frozen in liquid nitrogen and stored at − 80 °C until use. At least 15 mice were sacrificed at each time point and three independent samples were prepared.

### EVs from *E. falciformis*-infected mice serum

Blood samples were pooled and centrifuged within 1 h of collection by discontinuous centrifugation. The first centrifugation cycle was at 1500 *g* for 5 min, at room temperature, and the supernatant was transferred into a new tube and centrifuged at 2200 *g* for 10 min. A 5-ml sample of the serum was equilibrated with PBS (40%), and 3 µl of 0.05 M of EDTA was added per millilitre of the mixture, followed by centrifugation at 5000 *g* for 20 min, and 10,000 *g* for 30 min at 4 °C [30–32]. The resulting supernatant was ultrafiltered through 0.45-µm pore (Corning Inc., Corning, NY, USA) under gravity at 4 °C and then transferred into a polycarbonate tube for ultracentrifugation at 120,000 *g* for 18 h at 4 °C in a Sorvall™ wX+ Ultra Series Centrifuge with T890 fixed angle rotor: k-factor 25.1 (Thermo Fisher Scientific, Waltham, MA, USA). Finally, the pellet was collected and re-suspended in 1 ml of PBS (pH 7.2).

### EVs from *E. falciformis*-infected mice caecum

Extracellular vesicles were isolated from mice caecum as described previously [27–29, 34] with modifications. In brief, excised caeca were cut open on ice and the inflamed mucosal surface was scrapped with a microscope coverslip, pooled and homogenized on ice. A 1 g aliquot of the homogenate was added to 20 ml of papainase (w/v) (Solarbio, Beijing, China) and incubated for 20 min at 37 °C with intermittent stirring, followed by the addition of 200 µl of protease inhibitor (Solarbio, Beijing, China). The solution was centrifuged serially at 1500 *g* for 5 min, 2200 *g* for 10 min, 5000 *g *for 20 min and 10,000 *g* for 30 min, followed by ultrafiltration through a 0.45-µm microfilter (Corning Inc.) under gravity at 4 °C. Ultracentrifugation was performed under similar conditions as for the serum, and the pellet was re-suspended in 1 ml of PBS (pH 7.2).

### Purification of EVs on iodixanol density gradients

The resulting pellets from the ultracentrifugation were resolved on iodixanol gradient solutions prepared by diluting OptiPrep™ iodixanol (Sigma-Aldrich Norway, Merck Life Science AS, Oslo, Norway) stock solution into 60% (v/v) aqueous iodixanol with sterilized 0.3 M sucrose/10 mM Tris, pH 7.2. The gradient was formed by the sequential addition of 1.6 ml each of 40%, 20%, 10% and 5% (w/v) iodixanol preparation, and 1 ml of the ultracentrifuged pellet suspension was overlaid on the gradient. Differential ultracentrifugation was performed for 20 h at 120,000 *g* and 4 °C in a Sorvall wX+ Ultra Series Centrifuge (Thermo Fisher Scientific). Nine distinct gradient layers were collected (Additional file 1: Schematics), pooled, diluted 4 times with PBS (pH 7.2) and ultracentrifuged for 4 h at 120,000 *g* and 4 °C. The EV pellets were then pooled, concentrated in 100 µl of PBS and stored at − 80 °C. To determine the density of each gradient layer, a control iodixanol gradient with 1 ml of PBS was run in parallel and corresponding EV layers were collected and diluted 1:10,000 with PBS, and the optical density was measured at 244 nm by a UV–Visible spectrophotometer (Biomates 3S; Thermo Fisher Scientific) [30] (Additional file 1: Schematics).

### Nanoparticle tracking analysis

An equivalent volume of 10 μg each of sEVs and cEVs at each time point was diluted with PBS to make a total volume of 1 ml and measured in triplicate on a Zetasizer Nano-Zs instrument (Malvern Panalytical, Malvern, UK).

### Transmission electron microscopy

A 10-μl aliquot of EVs suspended in PBS was pipetted on Formvar copper grid (Znongjingkeyi, Beijing, China) for 3 min and then excess liquid was removed by a dry paper towel. The copper grid was washed with sterile PBS and immediately stained with 3% phosphotungstic acid (Solarbio, China) for 30 s and allowed to air-dry. Grids were imaged by transmission electron microscopy (TEM) using a Hitachi HT7700 (Hitachi Ltd., Tokyo, Japan).

### Protein digestion and tandem mass tag labelling

Protein quantification was performed at Lu-Ming Biotech Co., Ltd. (Shanghai, China). Protein concentration was determined using the Bicinchoninic Acid (BCA) Kit (Thermo Fisher Scientific). Briefly, 50 μg of protein was diluted by lysis buffer and adjusted to an equal concentration. Dithiothreito (DTT) was added to a final concentration of 5 mM and the protein preparation was incubated at 55 °C for 30 min. Subsequently, corresponding volume of iodoacetamide was added to make a final concentration of 10 mM. A sixfold volume of acetone was then added and the protein preparation kept at − 20 °C overnight, centrifuged at 8000 *g* for 10 min at 4 °C and the precipitate was collected. 100 μl of TEAB (200 mM) and 1 mg/ml of trypsin at 1:50 of the sample mass were added to reconstitute the pellet, followed by an overnight digestion at 37 °C. Thereafter, the sample was freeze-dried and stored at − 80 °C. Tandem mass tag (TMT) labelling was performed using the TMT pro16 (Thermo Fisher Scientific) with 20 μl of anhydrous acetonitrile, 10 μl of TMTpro16 reagent and 5 μl of 5% hydroxylamine were added. After 15 min, the solution was freeze-dried and stored at − 80 °C [31, 32].

### High-performance reverse-phase liquid chromatography

The labelled peptides were fractionated using a 1100 high-performance liquid chromatography (HPLC) Zorbax Extend-C18 Narrow Bore System (Agilent Technologies, Inc., Santa Clara, CA, USA). Mobile phase A contained 2% water and 98% acetonitrile while mobile phase B contained 90% acetonitrile and 10% water and were adjusted by ammonia to pH 10. The flow rate was set at 300 μl/min. Sample eluate was collected between 8 and 60 min and freeze-dried in vacuum for mass spectrometry.

### Liquid chromatography-tandem mass spectrometry analysis

Protein samples were transferred to pre-column Acclaim PepMap 100 HPLC columns (RP-C18; Thermo Fisher Scientific) at a flow rate of 300 nl/min and later separated by analytical column Acclaim PepMap RSLC RP-C18, (Thermo Fisher Scientific). The mobile phase A contained 99.9% water and 0.1% formic acid while the mobile phase B contained 80% acetonitrile, 19.9% water and 0.1% formic acid. The mass resolution of the primary MS, the automatic gain control and the maximum injection time was at 60,000, 1e6, 50 ms respectively. Mass spectrometry scan was set at m/z range of 350 to 1500. All MS/MS spectra were collected using high-energy collision fragmentation in data-dependent positive ion mode. The automatic gain control (AGC) was set to 2e5 while the maximum ion injection time was 45 min and the dynamic exclusion time was 60 s.

### Protein identification and bioinformatics

Raw protein sequences were searched against the *Mus musculus* Uniprot database (downloaded 12 Jan 2022) with Proteome Discoverer 2.4 (Thermo Fisher Scientific). The static mode was set as TMT (N-term, K), Carbamidomethyl (C) and the dynamic mode at Oxidation (M), Acetyl(N-term) in Orbitrap Fusion. MS1 and MS2 tolerance were 10 ppm and 0.02 Da, respectively. The database search was performed with trypsin digestion specificity with at most two missed cleavages. After the retrieval of original data, proteins were screened with Score Sequest HT > 0 and unique peptide ≥ Principal component analysis was performed using the expression of quantifiable proteins. Functional annotation for identified protein was performed using Proteome Discoverer 2.4. The relative peak intensities of TMT reporter ions released by tandem mass spectrometry (MS/MS) spectra were used. The fold change (FC) of differentially expressed proteins (DEPs) were determined as the mean of relative expression > 1.2 and < 0.85 for upregulated and downregulated proteins, respectively, with significance at *P* < 0.05. Functional annotation, including gene ontology (GO), at the levels of biological process, cellular component and molecular function (https://www.blast2go.com), as well as Kyoto Encyclopaedia of Genes (KEGG) (https://www.genome.jp/kegg/pathway.html) were also performed [32, 33]. PSORTb software was used to predict the subcellular localization of DEPs [34].

### Western blot

An equivalent volume of 10 µg of cEVs and sEVs at each time point was mixed separately with 4× Loading Buffer (Solarbio, Beijing, China), vortexed, and the mixture was placed in water bath at 100 °C for 10 min. The protein components were resolved in a 12% sodium dodecyl sulfate-polyacrylamide gel electrophoresis (SDS-PAGE) and then transferred to polyvinylidene difluoride membrane (Merck KGaA, Darmstadt, Germany). After 1 h of blocking in 0.01% PBST containing 5% skimmed milk, the membranes were incubated at 4 °C overnight with anti-Hsp70, anti-MHC, anti-CD9 and anti-CD82 (Proteintech Group, Rosemont, IL, USA). Thereafter, the membranes were incubated with horseradish peroxidase (HRP)-conjugated anti-rabbit antibodies (Proteintech Group) for 1 h at room temperature. The membranes were immersed in WesternBright™ enhanced chemiluminescence (ECL) solution (Advansta, San Jose, CA, USA) according to the manufacturer’s instruction and visualized using the Amersham Imager 600 chemiluminescence (Amersham Biosciences K.K., Tokyo, Japan).

### Statistical analyses

Wilcoxon signed rank tests was used for sampling time with an accepted significant level of *P* < 0.05. The sizes and subpopulations of EVs were expressed as the mean ± standard deviation and compared by the Welch t-test at significance levels of *P* < 0.05, *P* < 0.001 and *P* < 0.0001. Graphs were generated in GraphPad Prism 7 (GraphPad Software, San Diego, CA, USA).

## Results

### Morphological characterization of sEVs and cEVs

**Fig. 1:**
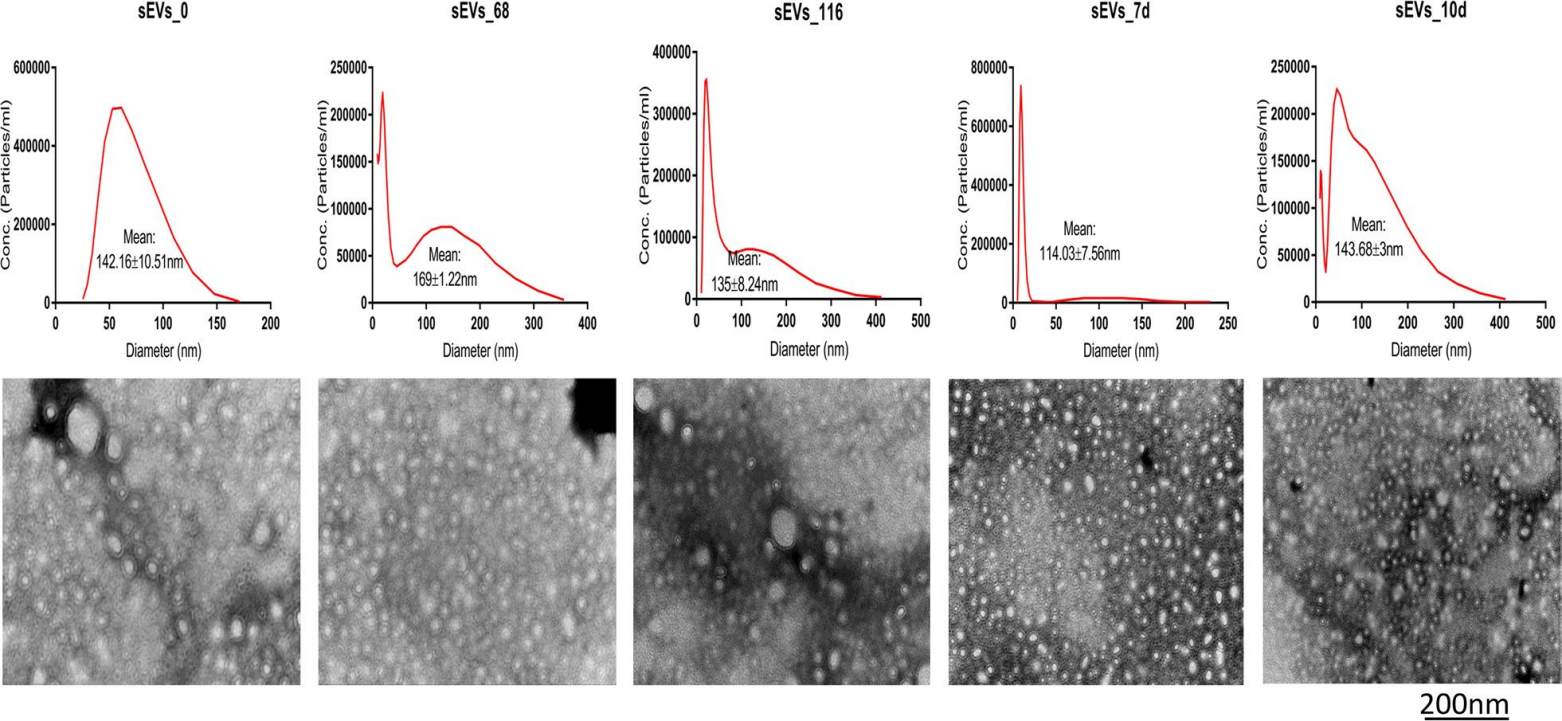
NTA and TEM analyses of sEVs isolated from *Eimeria falciformis* infected-mice during the early hours (< 1 hpi) of oocyst ingestion, first merozont generation (68 hpi), second merozont generation (116 hpi), latent period of oocyst shedding in the host faeces (7 dpi) and the onset of host recovery from the infection (10 dpi). An equivalent of 10 µg and 10 µl of sEVs was used for the NTA and TEM respectively. dpi, Days post-infection; hpi, hours post-infection; NTA, nanoparticle tracking analysis;sEVs, serum-derived extracellular vesicles; TEM, transmission electronic microscopy

**Fig. 2:**
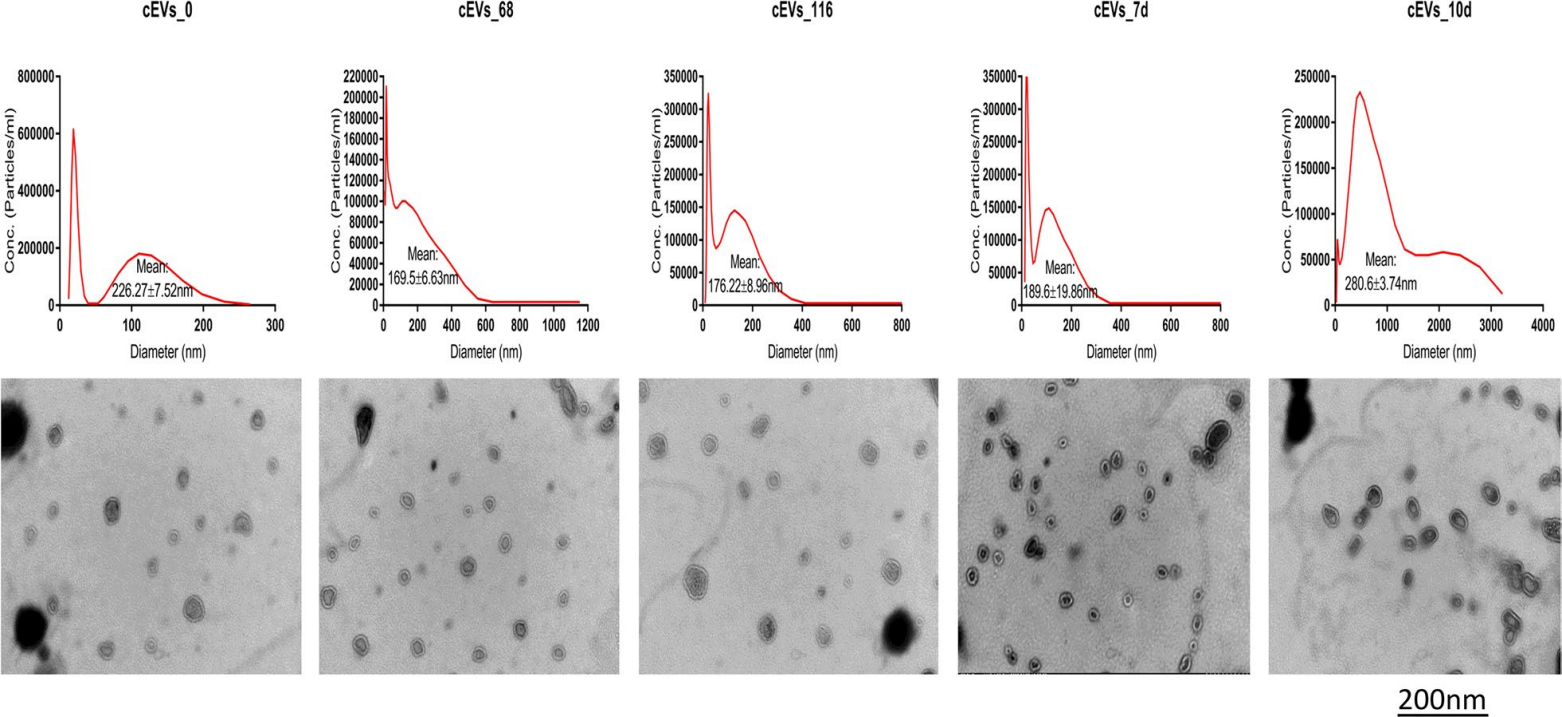
NTA and TEM analyses of caecum-derived EVs (cEVs) isolated from *E. falciformis* infected-mice during the early hour (< 1 h) of oocyst ingestion, first merozont generation (68 hpi), second merozont generation (116 hpi), latent period of oocyst shedding in the host faeces (7 dpi) and the onset of host recovery from the infection (10 dpi). An equivalent of 10 µg and 10 µl of cEVs was used for the NTA and TEM respectively. cEVs, Caecum-derived extracellular vesicles; dpi, days post-infection; hpi, hours post-infection; NTA, nanoparticle tracking analysis; TEM, transmission electronic microscopy

**Fig. 3:**
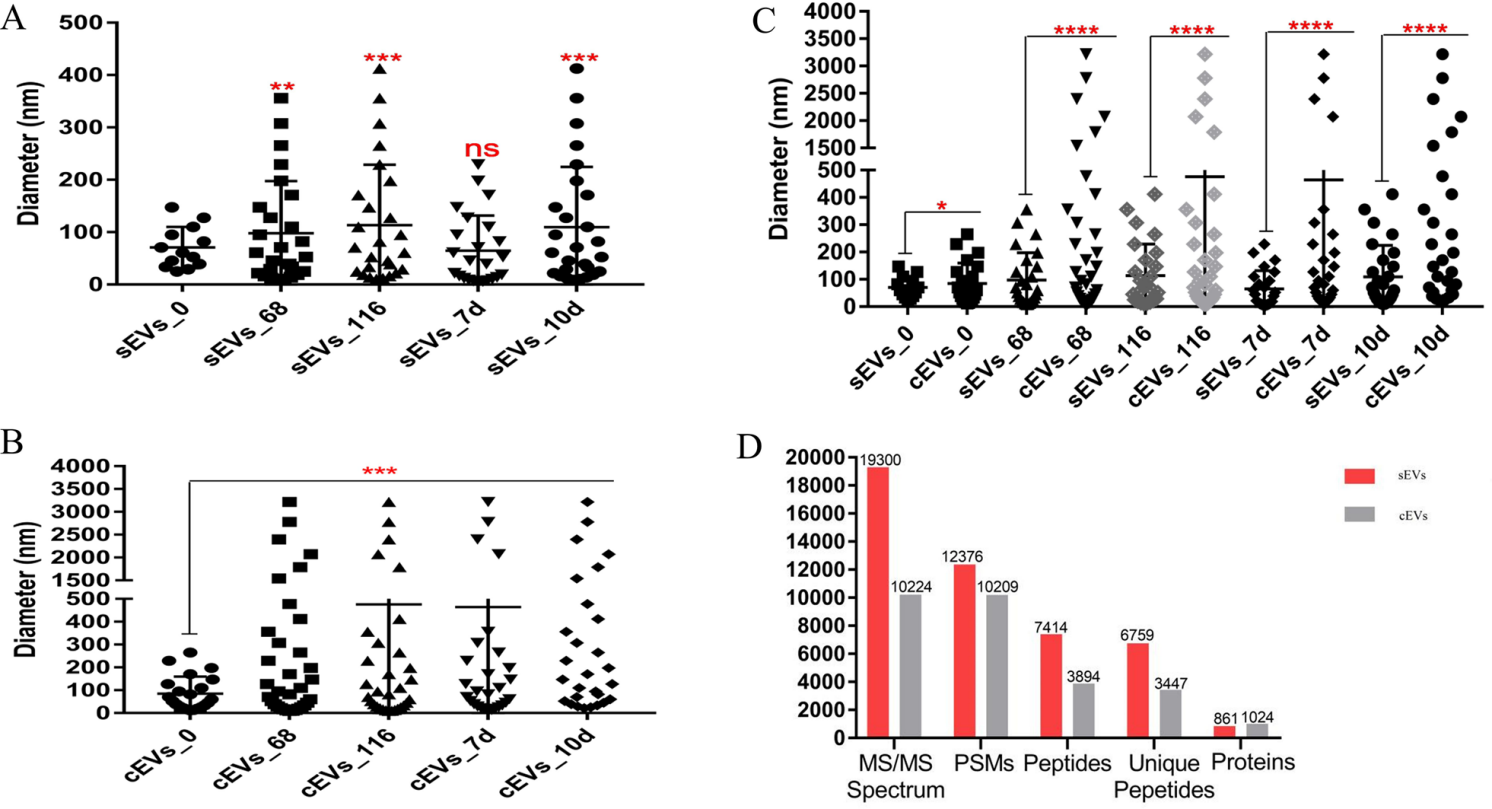
Subpopulations of sEVs and cEVs **a** comparison of sEV subpopulations during stages of the parasite infectious cycle, **b** comparison of cEV subpopulations during stages of the parasite infectious cycle, **c** comparison of sEV and cEV subpopulations during the parasite infectious cycle, **d** MS/MS peptide spectra of cEVs and sEVs. Asterisks indicate a significant difference at *P<0.05, **P<0.001, ***P<0.0001 and ****P<0.00001; ns, not significant. cEVs, Caecum-derived extracellular vesicles; MS/MS, tandem mass spectrometry; PSM, peptide spectra match; sEVs, serum-derived extracellular vesicles

Nanoparticle tracking analysis (NTA) showed that sEV subpopulation had considerably increased in number and size from 68 hpi to 10 dpi and the distribution was heterogenous (Fig. 1). The sEVs ranged in size from 9.08 to 412.56 nm, and TEM analysis revealed that they have a double membrane and are nearly spherical in shape (Fig. 1). In comparison, TEM revealed that cEVs were also heterogenous in shape (Fig. 2), and ranged from 9.08 to 3219 nm in size (Fig. 2) and increased in number between 68 hpi and 10 dpi (Fig. 3a, b). This latter increase suggests that *E. falciformis* triggered the formation of additional subpopulations of EVs at the local and systemic levels. In addition, there was a significantly higher number of subpopulations of cEVs than of sEVs (Fig. 3c) and the number of subpopulations of cEVs and sEVs at 68 hpi to 10 dpi were significantly higher that the number of EV subpopulations at 0 hpi (Fig. 3c). Also, the densities of isolated EVs were between 1.101 and 1.215 g/cm^3^ (Additional file 1: Schematics).

### EV proteins and markers

**Fig. 4:**
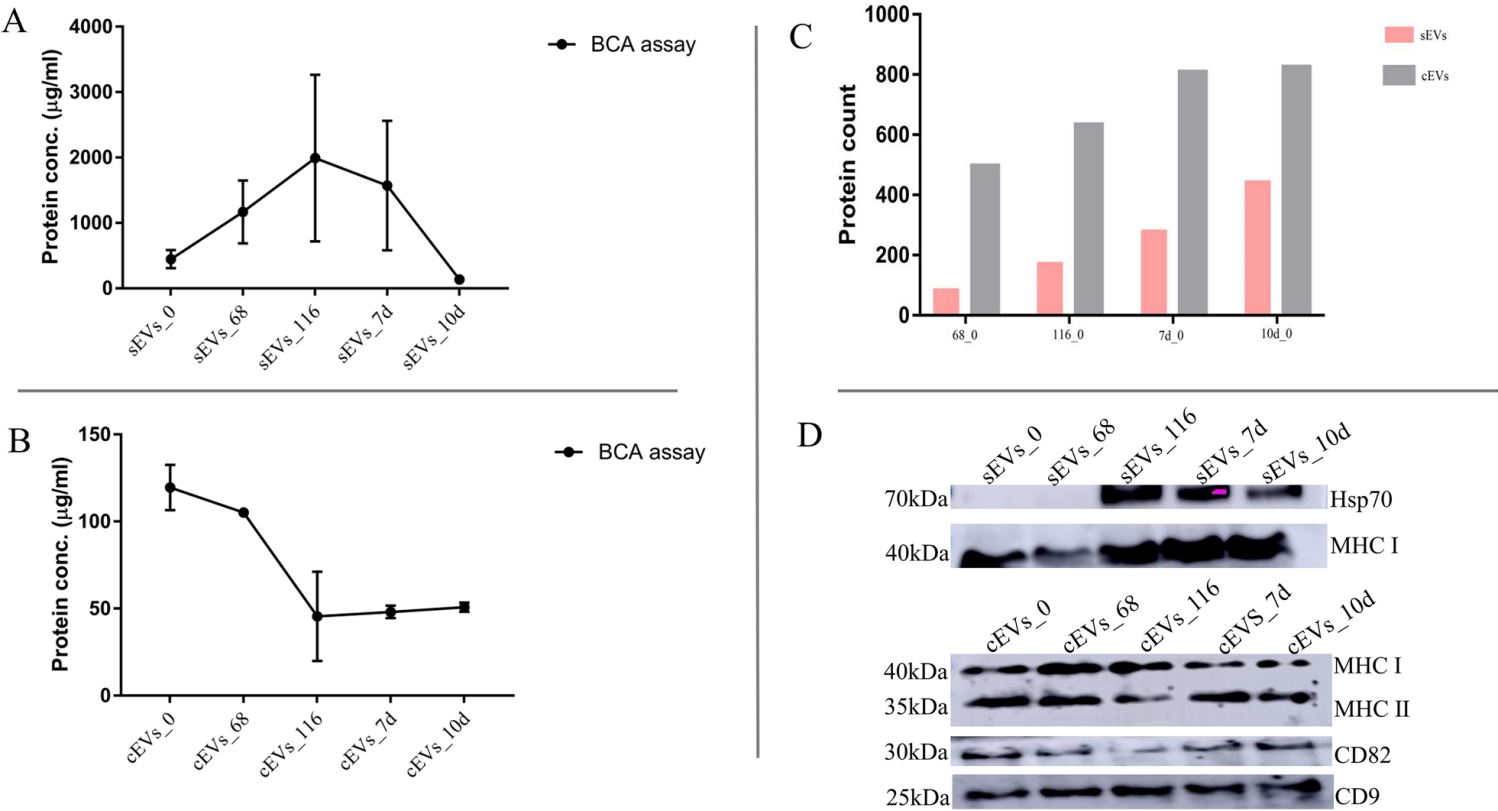
Quantification and identification of EV proteins.** a** BCA analysis of sEV proteins, **b** BCA analysis of cEV proteins, **c** sEV and cEV protein count, **d** Western blot analyses of sEV and cEV protein markers. BCA, Bicinchoninic acid; CD9, CD82, MHC I and II, Hsp70 Molecule cEVs, caecum-derived extracellular vesicles; Hsp70, heat shock protein 70; MCH, major histocompatibility complex; sEVs, serum-derived extracellular vesicles

The HPLC–MS/MS analysis identified a total of 19,300 and 10,224 MS/MS spectra in sEVs and cEVs respectively, as well as 12,376 and 10,209 peptide spectra match (PSMs) in sEVs and cEVs, respectively (Fig. 3d) (Additional file 2:). The MS secondary spectrograms for sEVs and cEVs were 64.12% and 99.85%, respectively. A total of 7414 and 3894 peptides were identified by spectrographic analysis, of which 6759 and 3447 peptides were from sEVs and cEVs, respectively (Fig. 3d; Additional file 3; Additional file 4). In all, 860 and 1024 quantifiable proteins were identified in sEVs and cEVs, respectively (Fig. 4c) (Additional file 4; Additional file 5) while 35 proteins were common to both cEVs and sEVs (Additional file 1). The protein distributions across the parasite stages in the host are shown in a Venn diagram (Additional file 1).

There are at least 200 EV markers [35]. In this study, MHC I was found to be common to both cEVs and sEVs. cEVs also expressed CD9, CD82 and MHC II whereas Hsp70 was common in sEVs across the *E. falciformis* stages examined and confirmed by western blot (Fig. 4d). Other sEV markers identified include Annexin 7, an apoptotic marker, at 68 and 116 hpi as well as H2A, H3, H4 and Hsp90 (Additional file 6). Other identified cEV markers include CD63, Hsp90, CD151, histones and Annexin A1-6. Identification of Annexin 7 at 68 and 116 hpi signifies that *E. falciformis* merozont formation is accompanied by host cell death (Additional file 7). Also, cEVs enclosed ESCRT (endosomal complexes required for transport)-associated, vacuolar protein sorting that participates in the release of EVs and charged multivesicular body (MVB) protein 3 that harmonizes transmembrane proteins into lysosomes/vacuoles via the MVB pathway at 7 and 10 dpi (Additional file 7).

### Differential expression analyses

**Fig. 5:**
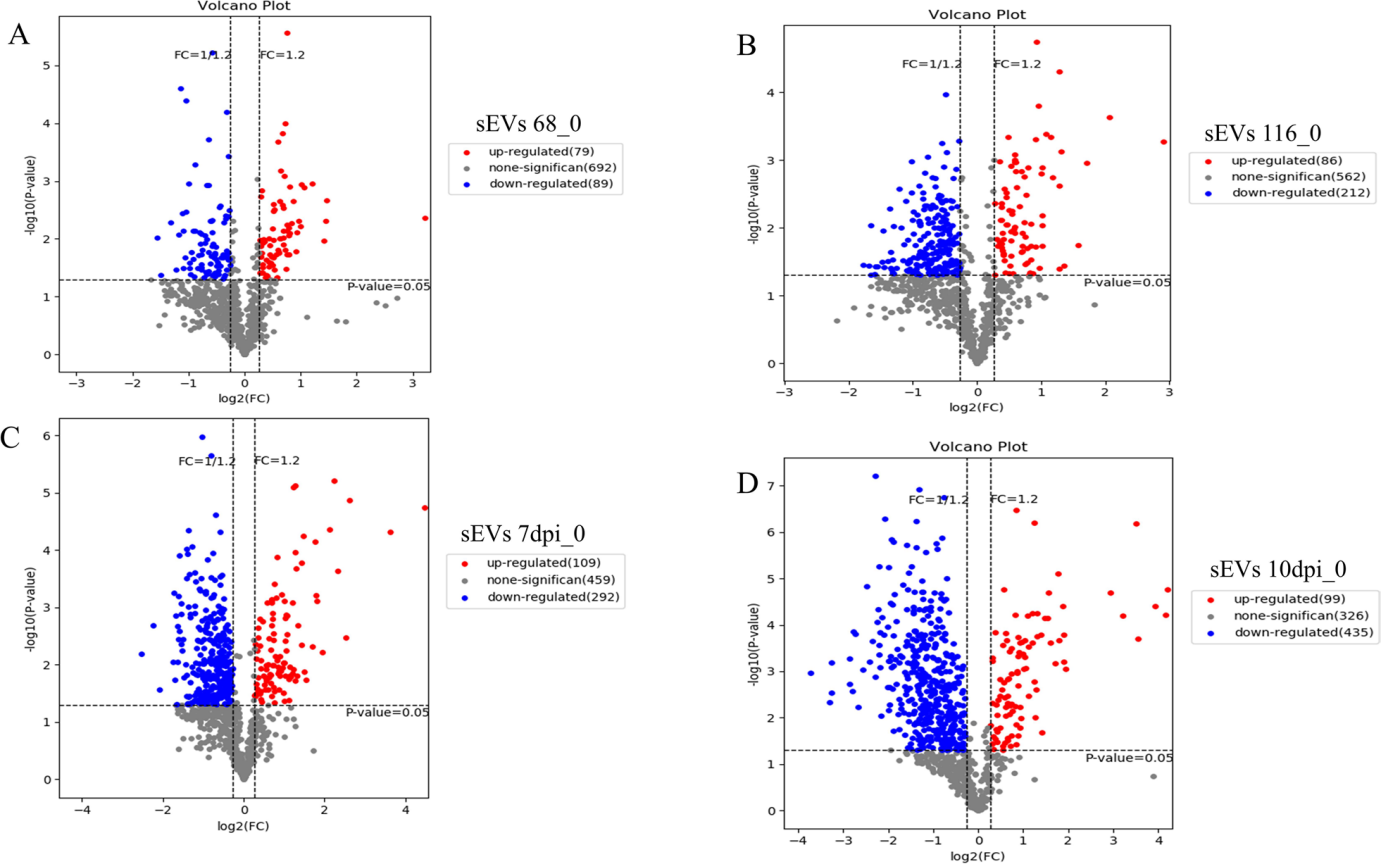
Differentially expressed sEV proteins. Identified proteins from sEVs across the parasite infectious cycle were analyzed using a binary differential comparison between time of *E. falciformis* oocyst ingestion by the host (<1 hpi) and first merozont stage (**a**; 68 hpi), second merozont stage (**b**; 116 hpi), *E. falciformis* oocyst shedding (**c**; 7 dpi) and onset of host recovery from *E. falciformis* infection (**d**; 10 dpi). FC > 1.2 was taken as indicating upregulation and FC < 0.85 was taken as indicating downregulation. dpi, Days post-infection; FC, fold change; hpi, hours post-infection; sEV, serum-derived extracellular vesicle

**Fig. 6:**
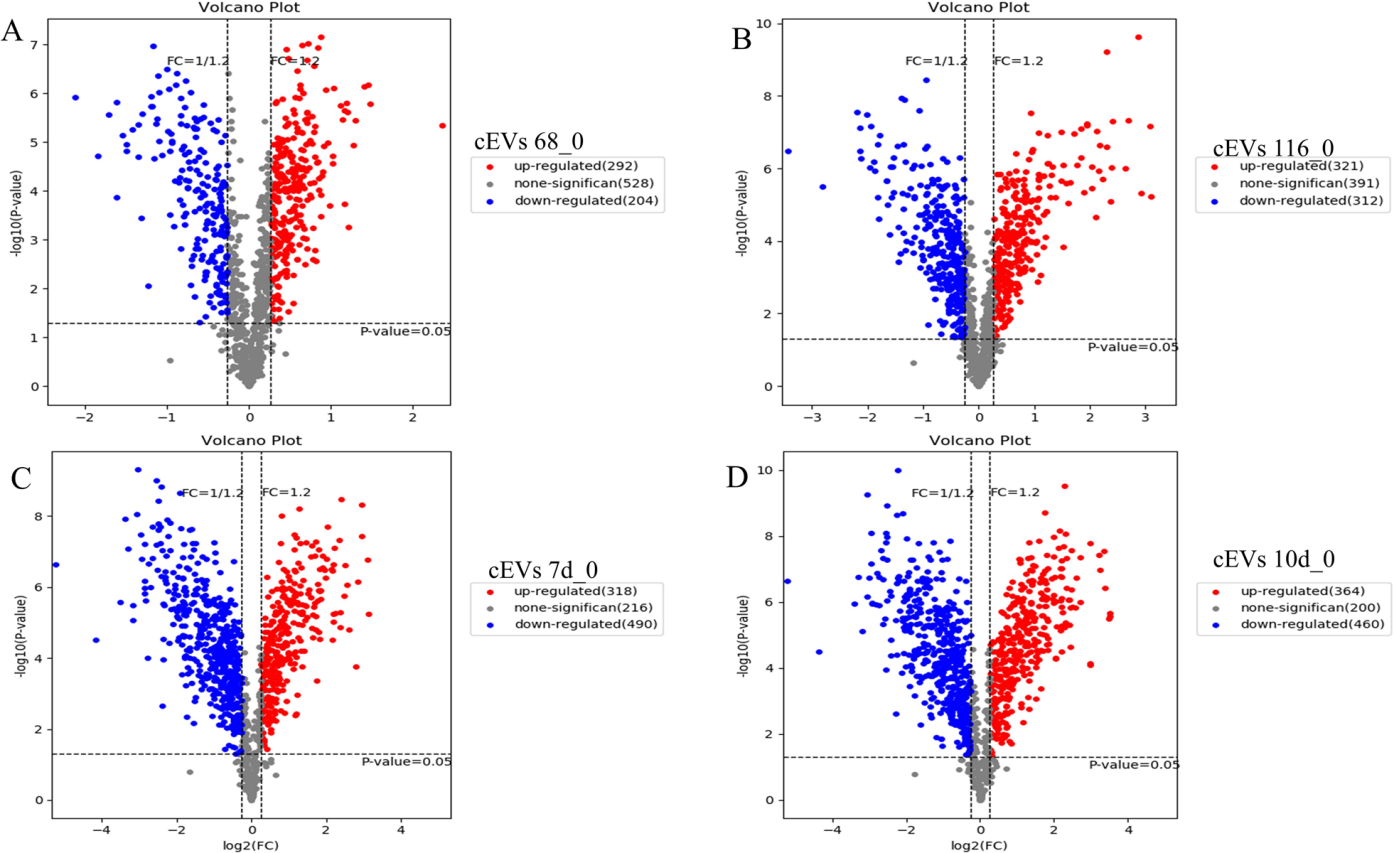
Differentially expressed cEV proteins. Identified proteins from cEVs across the parasite infectious cycle were analyzed using a binary differential comparison between time of *E. falciformis* oocyst ingestion by the host (< 1 h) and first merozont stage (**a**; 68 hpi), second merozont stage (**b**; 116 hpi), *E. falciformis* oocyst shedding (**c**; 7 dpi) and onset of host recovery from *E. falciformis* infection (**d**; 10 dpi). FC > 1.2 was taken as indicating upregulation and FC < 0.85 was taken as indicating downregulation. cEV, caecum-derived extracellular vesicle; dpi, days post-infection; FC, fold change; hpi, hours post-infection

Having observed that protein expressions differed considerably (Additional file 3, Additional file 4), a binary differential comparison was used to compare EV proteins at 0 hpi with those at other time points using a 1.2-FC for DEPs (Fig. 5). sEVs enclosed 79 upregulated and 89 downregulated proteins between oocyst ingestion (0 hpi) and the first merozont stage (68 hpi). Also, 86 and 212 sEV proteins were upregulated and downregulated, respectively, at the *E. falciformis* second merozont stage (116 hpi). At 7 dpi, 109 sEV proteins were upregulated and 292 were downregulated, indicating an increased number of sEV proteins at the onset of oocyst shedding. By 10 dpi, 95 proteins were upregulated and 435 were downregulated (Fig. 5) (Additional file 6). In cEVs, 292 proteins were upregulated while 204 proteins were downregulated, respectively, at 68 hpi. At 116 hpi, 321 and 312 proteins were up- and downregulated, respectively, in cEVs. By 7 dpi, 318 and 490 proteins were up- and downregulated, respectively, in cEVs (Fig. 6). As host recovered from infection began (10 dpi), 364 and 460 cEV proteins were up- and downregulated, respectively (Additional file 6).

### EV proteins associated with immunity and host cell death

Differentially regulated sEV immune-associated proteins are diverse and include proteins associated with neutrophil, monocytes, complements, interleukin, chemokine as well as interferon, receptor and cell death-associated proteins (Table 1) (Additional file 5). cEV-bound proteins associated with cell death were upregulated (Table 2). Lymphocyte antigen, caspase 3 and signal transducer and activator of transcription 1 (STAT1) were either downregulated or undetected while leukocyte surface antigen and neutrophilic granule were upregulated as the host recovered from the infection and during the latent period of oocyst shedding (Table 2). However, transforming growth factor beta (TGF-β), immunoglobulins (Ig) and toll-like receptor-associated proteins remained significantly downregulated across the parasite developmental stages in the host (Table 2) (Additional file 6).

**Table 1:** Differentially expressed immune- and cell death-associated protein in serum-derived extracellular vesicles

Protein ID	Gene name	Protein description	Coverage (%)	Unique peptides (*n*)	Sequence alignment score	FC			
						sEVs, 0/68 hpi	sEVs, 0/116 hpi	sEVs, 0/7 dpi	sEVs, 0/10 dpi
P04223	H2-K1	H-2 class I histocompatibility antigen	10	2	34.8	1.54	2.11	1.67	
P14426	H2-D1	H-2 class I histocompatibility antigen	15	2	57.72	1.65	2.22	1.51	0.6
P11672	Lcn2	Neutrophil gelatinase-associated lipocalin	26	5	25.89	ND	2.01	4.35	2.38
P08607	C4bpa	C4b-binding protein	36	15	269.56	ND	2.27	2.02	1.62
Q9Z0M9	Il18bp	Interleukin-18-binding	6	1	8.12	ND	1.64	ND	0.62
P10810	Cd14	Monocyte differentiation antigen CD14	19	6	28.9	ND	ND	2.69	ND
Q9Z121	Ccl8	C-C motif chemokine 8	59	4	21.2	ND	ND	1.61	ND
Q9ESY9	Ifi30	Gamma-interferon-inducible lysosomal thiol reductase	4	1	3.03	ND	1.5	ND	ND
Q61730	Il1rap	Interleukin-1 receptor	16	8	12.54	ND	0.52	0.52	0.59
O08688	Capn5	Calpain-5 accessory protein	25	16	158.62	0.61	0.52	0.4	0.22
P12399	Ctla2a	Protein CTLA-2-alpha	26	3	9.88	0.61	0.5	0.52	0.23
P70445	Eif4ebp2	Eukaryotic translation initiation factor 4E-binding	5	1	2.01	0.48	0.47	0.42	0.15
Q9WU78	Pdcd6ip	Programmed cell death 6-interacting protein	14	11	24.09	ND	0.59	0.61	0.54
Q8CJ96	Rassf8	Ras association domain- containing protein 8	3	1	7.08	ND	0.56	0.33	0.18
P06683	C9	Complement component C9	59	29	267.09	0.45	ND	0.49	0.2
Q01279	Egfr	Epidermal growth factor receptor	25	27	207.31	0.63	ND	0.57	0.28
P09581	Csf1r	Macrophage colony-stimulating factor 1 receptor	11	8	48.61	ND	ND	ND	0.43
P29533	Vcam1	Vascular cell adhesion	12	7	24.93	ND	ND	ND	0.42

*dpi* Days post-infection,* FC* fold change,* hpi* hours post-infection, *ND *not determined,* sEVs* serum-derived extracellular vesicle

**Table 2:** Differentially expressed immune- and cell death-associated proteins in caecum-derived extracellular vesicles

Protein ID	Gene name	Protein description	Coverage (%)	Unique peptides (*n*)	Sequence alignment score	FC			
						cEVs, 0/68 hpi	cEVs, 0/116 hpi	cEVs, 0/7 dpi	cEVs, 0/10 dpi
P12815	Pdcd6	Programmed cell death protein 6	24	4	32.02	1.32	1.42	3.07	2.29
Q9JJF9	Sppl2a	Signal peptide peptidase-like 2A	5	4	34.43	1.57	0.79	2.16	3.13
P14437	H2-Aa	H-2 class II histocompatibility antigen	22	1	44.04	2.24	1.62	1.63	1.62
P01901	H2-K1	H-2 class I histocompatibility antigen	14	1	58.31	1.42	1.76	2.33	3.65
P29477	Nos2	Nitric oxide synthase, inducible	4	4	20.91	1.24	ND	1.32	1.29
Q9QZ85	Iigp1	Interferon-inducible GTPase 1	6	2	11.66	2.03	2.32	1.98	1.98
Q91WS2	Nlrp6	NACHT,LRR and PYD domains-containing protein 6	3	2	5.21	1.12	0.77	1.66	2.67
P05533	Ly6a	Lymphocyte antigen 6A-2/6E-1	16	1	35.53	1.52	1.21	1.36	0.69
O08692	Ngp	Neutrophilic granule protein	12	2	16.55	1.36	ND	7.79	2.76
P42225	Stat1	Signal transducer and Activator of transcription	11	6	5.58	1.48	2.43	2.2	ND
Q61735	Cd47	Leukocyte surface antigen CD47	16	4	26.63	ND	0.77	1.23	1.68
P70677	Casp3	Caspase-3	14	4	25.62	ND	1.58	1.21	0.76
Q08509	Eps8	Epidermal growth factor Receptor kinase substrate 8	11	9	57.71	0.63	0.69	1.94	2.67
O88456	Capns1	Calpain small subunit	3	1	5.77	1.26	1.62	1.68	1.48
Q99JW5	Epcam	Epithelial cell adhesion molecule	21	5	89.87	0.63	1.22	0.46	0.49
P01878		Ig alpha chain C region	11	3	39.71	0.57	0.45	0.29	0.19
Q2KHK6	Gsdmc2	Gasdermin-C2	6	3	15.83	0.7	0.58	0.29	0.32
P82198	Tgfbi	Transforming growth factor-beta-induced protein ig-h3	1	1	2.8	0.83	0.62	ND	0.37
P18531	Ighv3-6	Ig heavy chain V region 3-6	17	1	2.8	0.62	0.69	0.51	0.3
P11438	Lamp1	Lysosome-associated membrane glycoprotein 1	6	2	21.15	0.83	0.7	0.64	0.83
P58682	Tlr8	Toll-like receptor 8	1	1	2.92	ND	0.57	0.15	0.17
Q3T9E4	Tgtp2	T-cell-specific guanine nucleotide triphosphate-binding protein	1	1	4.64	ND	0.62	0.32	0,61
P01831	Thy1	Thy-1 membrane glycoprotein	9	1	3.13	ND	0.75	0.54	0.6

* cEVs* Caecum-derived extracellular vesicles, *dpi* days post-infection,* FC* fold change,* hpi* hours post-infection

Comparison of sEV and cEV DEPs showed that corresponding immune protein candidates remained upregulated until *E. falciformis* second merozont stage (116 hpi) and thereafter were downregulated (Additional file 5, Additional file 6). Caecum-derived EVs contained significantly upregulated complement proteins downregulated in sEVs. Ig-associated proteins were upregulated in sEVs but completely downregulated in cEVs across the infectious cycle except for transmembrane Ig domain (Additional file 6). In addition, isotypes of class I and II histocompatibility antigens in sEVs were significantly upregulated across the major stages of the *E. falciformis* infectious cycle (Fig. 4; Additional file 6). Specifically, CD5 antigen-like was found throughout the infectious cycles of the parasites and could have undergone transcytosis through the blood stream and inflamed caecum from the spleen, lymph nodes or thymus (Table 3).

**Table 3:** Differentially expressed immune-related proteins in serum- and caecum-derived extracellular vesicles

Protein ID	Gene name	Protein description	Coverage (%)	Unique peptides (*n*)	Sequence alignment score	FC				
						sEVs/cEVs, 0 hpi	sEVs/cEVs, 68 hpi	sEVs/cEVs, 116 hpi	sEVs/cEvs, 7 dpi	sEVs/cEVs, 10 dpi
P01902	H2-K1	H-2 class I histocompatibility antigen	14	1	58.31	2.18	1.66	1.23	1.58	0.27
P82198	Tgfbi	Transforming growth factor-beta-induced protein ig-h3	88.8	128.5	2.8	0.75		1.56	0.71	1.45
Q9WU78	Pdcd6ip	Programmed cell death 6-interacting protein	173.9	50.4	143.72	2.77	1.5	1.44	0.51	0.61
P01027	c3	Complement C3	68.9	35	35.25	2.23	3.44	2.58	0.38	
P63001	Rac1	Ras-related C3 botulinum toxin substrate 1	184.9	34.8	12.75	4	1.78	1.36	0.8	2.58
P13597	Icam1	Intercellular adhesion molecule 1	102.4	79.6	11.99		1.21		1.44	0.42
P01029	C4b	Complement C4-B	97	62.4	28.46		1.57	1.95	0.65	0.64
P01631		Ig kappa chain V-II region 26-10	85.2	43.2	23.02	2.35	2.01	1.29	0.45	0.75
Q9QWK4	Cd5l	CD5 antigen-like	83.4	49.2	5.27	1.47	1.6	1.9	0.68	0.77
P11672	Lcn2	Neutrophil gelatinase-associated lipocalin	50.1	14.2	6.02	1.97	3.47	4.22		0.46

* cEVs* Caecum-derived extracellular vesicles, *dpi* days post-infection,* FC* fold change,* hpi* hours post-infection,* sEVs* serum-derived extracellular vesicles

, 

### EVs proteins associated with metabolism and ion transport

Some EV proteins involved in metabolism and ion transport found in sEVs include zinc finger domain and associated protein, which regulate RNA metabolism and interact with stress-related proteins, and plasma membrane calcium-transporting ATPase, which actively transports calcium from cytoplasm into the extracellular space (Table 4). However, extracellular superoxide dismutase, which scavenges the superoxide anion known to prevent oxidative stress and damage in a variety of disease pathologies, was found significantly low in sEVs (Table 4). Other downregulated metabolic proteins in sEVs are listed in Table 4 and Additional file 5: Table S5. In contrast, in cEvs, calcium-activated chloride channel, pyruvate kinase, Nck-associated and eukaryotic initiation factor proteins were upregulated throughout the infection time points (Table 4). Downregulated ion-transport proteins in cEVs include cytochrome* c* oxidase, and chloride- and calcium ion-associated proteins (Table 5; Additional file 7).

**Table 4:** Differentially expressed homeostatic and ion exchange proteins in serum-derived extracellular vesicles

Protein ID	Gene name	Protein description	Coverage (%)	Unique peptides (*n*)	Sequence alignment score	FC			
						sEVs, 0/68 hpi	sEVs, 0/116 hpi	sEVs, 0/7 dpi	sEVs, 0/10 dpi
Q64518	Atp2a3	Sarcoplasmic/endoplasmic reticulum calcium ATPase 3	7	3	15.26	ND	0.4	0.41	ND
P52480	Pkm	Pyruvate kinase PKM	53	23	ND	ND	ND	0.66	0.56
Q64726	Azgp1	Zinc-alpha-2-glycoprotein	49	18	190.76	ND	0.66		0.38
P68254	Ywhaq	14-3-3 protein theta	31	3	34.47	ND	0.6	0.6	0.47
P08228	Sod1	Superoxide dismutase [Cu-Zn]	37	6	27.49	ND	0.55	0.57	ND
Q6X893	Slc44a1	Choline transporter-like protein 1	2	1	5.15	ND	0.55	0.51	0.42
P24668	M6pr	Cation-dependent mannose-6-phosphate receptor	8	1	2.89	ND	0.52	ND	ND
Q8CHP0	Zc3h3	Zinc finger CCCH domain-containing protein 3	1	1	2.21	ND	1.63	1.63	ND
O09164	Sod3	Extracellular superoxide dismutase [Cu-Zn]	29	5	35.01	ND	ND	0.43	0.27
Q60932	Vdac1	Voltage-dependent anion-selective channel protein	10	2	7.01	ND	ND	0.63	0.23
Q6Q477	Atp2b4	Plasma membrane Calcium- transporting ATPase 4	1	1	4.22	ND	ND	ND	1.89

*dpi* Days post-infection,* FC* fold change,* hpi* hours post-infection,* sEVs* serum-derived extracellular vesicles

**Table 5:** Differentially expressed homeostatic and ion exchange proteins in caecum-derived extracellular vesicles

Protein	Gene name	Protein description	Coverage (%)	Unique peptides (n)	Sequence alignment score	FC			
						cEVs, 0/68 hpi	cEVs, 0/116 hpi	cEVs, 0/7 dpi	cEVs, 0/10 dpi
Q9CQZ6	Ndufb3	NADH dehydrogenase[ubiquinone] 1 beta subcomplex subunit	11	1	2.96	0.701	0.7	0.26	0.34
P52480	Pkm	Pyruvate kinase PKM	14	8	89.8	1.36	2.05	1.38	1.69
P28660	Nckap1	Nck-associated protein 1	4	5	27.17	1.29	1.39	2.23	1.7
P60843	Eif4a1	Eukaryotic initiation factor 4A-I	11	6	23.68	1.33	1.56	1.33	1.36
Q9WVC8	Slc26a3	Chloride anion exchanger	5	4	31.46	1.45	0.43	0.26	0.5
Q6Q473	Clca4a	Calcium-activated chloride channel regulator 4A	4	4	35	1.45	ND	1.4	5.55
O08532	Cacna2d1	Voltage-dependent calcium channel subunit alpha-2/delta-1	6	5	12.7	0.77	0.48	ND	0.56
P62908	Rps3	40S ribosomal protein S3	18	4	20.04	1.02	0.71	0.31	0.26
P54071	Idh2	Isocitrate dehydrogenase [NADP] mitochondrial	5	3	18.07	1.54	1.58	1.32	0.68
Q8BXK9	Clic5	Chloride intracellular channel protein 5	19	3	13.41	ND	0.75	0.8	ND
Q3UVU3	Slc30a10	Zinc transporter 10	2	1	5.35	1.35	0.37	0.59	0.59
O55143	Atp2a2	Sarcoplasmic/endoplasmic reticulum calcium ATPase 2	1	1	8.69	ND	0.8	0.65	2.31
P19536	Cox5b	Cytochrome c oxidase subunit 5B	9	1	6.6	0.37	0.53	0.28	0.24

* cEVs* Caecum-derived extracellular vesicles, *dpi* days post-infection,* FC* fold change,* hpi* hours post-infection

### GO and KEGG analyses

The GO functional annotation for sEV proteins (Additional file 7) and cEVs (Additional file 8) were analyzed by the Blast2GO bioinformatics platform. The GO top 5 hits of sEV proteins revealed that they are components of extracellular space/regions and matrix. At the first *E. falciformis* merozont stage (68 hpi), catabolic processes and negative regulation of gluconeogenesis were top hits. At the *E. falciformis* second merozont stage (116 hpi), sEV proteins were involved in complement activation, cytokines and hormonal responses. During oocyst shedding (7 dpi), catabolic activities and endopeptidase ranked at the top of GO terms for sEV proteins. At 10 dpi, top GO functions of sEV proteins were related to blood coagulation, complement activation and negative activation of endopeptidase activities (Additional files 1, 7).

GO functional analyses of cEV proteins indicated catabolic process, ion transport and antigen presentation during the first and second merozont stages (68 and 116 hpi, respectively). At the time of oocyst shedding, response to intestinal bacterium, localization of plasma membrane and catabolic process were predominant. During host recovery (10 dpi), cell adhesion and intercellular protein transport ranked high. cEV proteins possibly originated from the (apical) plasma membrane, myelin sheath, brush boarder, cytosol and cellular matrix. Annotated molecular functions of cEV proteins include endopeptidase activities and calcium ion binding, especially during oocyst shedding (7 dpi) and host recovery (10 dpi) (Additional file 1, Additional file 8).

The roles of the identified proteins were determined using the online KEGG database. Among the first top 20 hits for sEV proteins were complement and coagulation cascade, antigen processing and presentation across *E. falciformis* major developmental stages in the host (Additional file 9). Also, the peroxisome proliferator-activated receptor (PPAR) and Fc-gamma R-mediated phagocytosis pathways were associated with sEV proteins during the formation of first and second merozonts. Importantly, the NK cell-mediated cytotoxicity pathway was enriched during the *E. falciformis* first merozont stage, indicating an early innate host response. Leukocyte trans-endothelial migration and apoptosis pathways were observed at the time of oocyst shedding (7 dpi) (Additional file 1). Ferroptosis, Th1 and Th2 cell differentiation and Th17 pathways were also observed at the time of host recovery (Additional file 9), indicating intracellular iron-dependent host cell death and late response of cellular immunity during *Eimeria* infection, respectively.

Similarly, the first top 20 hit KEGG pathways for cEV proteins include antigen processing and presentation, and graft-versus-host disease throughout the *E. falciformis* infectious cycle in the host. Ferroptosis and necroptosis (regulated inflammatory cell death) were found across the infectious cycle whereas apoptosis (at first merozont), leukocyte trans-endothelial migration, toxoplasmosis and viral carcinogenesis coincided with the time of first and second merozont formation (Additional file 1, Additional file 10) in the host. cEV proteins during the *E. falciformis* second merozont stage (116 hpi), time of oocyst production and host recovery were associated with the interleukin-17 (IL-17), Th17 cell differentiation, NOD-like receptor and hypoxia-inducible factor-1 (HIF-1) signaling pathways. Protein digestion and absorption as well as regulation of actin cytoskeleton pathways were common during oocyst shedding and host recovery from *E. falciformis* infection. Only at the time of host recovery (10 dpi) was the cell adhesion molecule pathway found for cEV proteins (Additional file 10).

## Discussion

The host-parasite interaction is a complex and dynamic phenomenon, especially for monoxenous parasites such as *Eimeria* species whose developmental transitions are associated with the obligatory triggering of diverse host responses. In the present study, we performed comparative proteomic analysis of local and systemic host circulating EVs with the aim to obtain a broader insight into the dynamics of host responses to *E. falciformis* infection during parasite development and/or interaction with the host. EVs were isolated from the blood serum and caecal mucosa layer of *E. falciformis*-infected mice within few minutes after oral inoculation of (sporulated) oocysts (0 hpi) and during the first merozont stage (68 hpi), second merozont stage (116 hpi), oocyst shedding (7 dpi) and at the onset of host recovery (10 dpi).

The shape, size and subpopulation of EVs derived from *E. falciformis* infected-mice serum and caecum increased significantly with the progression of the parasite infection (Figs. 1, 2, 3). In previous investigations, significant numbers of microparticles were observed in *Plasmodium-*infected humans [36], *Plasmodium vivax*-infected individuals [37] and *Toxoplasma gondii*-infected mice [38]. During *E. falciformis* infection, IECs as potential antigen-presenting cells (APCs) have been reported to capture parasite antigens or EVs [30], resulting in activation and allostimulation of infected/uninfected cells to secrete more vesicles, as reported in *Giardia intestinalis* and *Plasmodium yoelli* [39, 40]. It has also been shown that the resulting enteric lesion, epithelial sloughing and focal hyperplasia of epithelium from *Eimeria* infection [41] may cause leaky caecal endothelium with the secretion of EVs. In addition, epithelial-mesenchymal transition due to infection is known to support exosome secretion [42] in association with epithelial destruction and apoptotic changes during *E. intestinalis* infection [22]. Also, intraepithelial or circulatory immune cells may secrete EVs that infiltrate inflamed caecum in response to *E. falciformis* infection. More importantly, *Eimeria*-host cell interaction could lead to massive destruction of absorptive and cryptal intestinal epithelial cell as well as submucosal oedema [43] with concomitant secretion of EVs. Summarily, the results of this study indicate that additional host-derived, local and systemic EVs are secreted as *E. falciformis* undergoes developmental transitions and/or interacts with host IECs.

The LC–MS/MS results indicated that there was considerable variation in EV proteins during the course of infection. Among the cEV markers identified in this study is CD9, which was also found in plasma-derived exosome from chimeric humanized mice infected with *P. vivax* [23] and in Balb/c mice thymocyte-derive microvesicles [44]. In addition, class I and II histocompatibility antigens (Fig. 4) and Annexin (A1-6) as well as other classical exosome markers reported in mouse model of malaria infection, such as glyceraldehyde 3-phosphate dehydrogenase (GAPDH) and Ras-related proteins [23] and human IEC exosome-like MHC I and II and CD63 [45] were identified in this study (Additional file 5, Additional file 6), attesting to the vesicular properties, in addition to the densities (Additional file 1: Figure S1) of cEVs and sEVs.

MHC molecules and CD5 are known to play pivotal roles in the cell-mediated immune system and APCs [44]. Specifically, MHC II from exosomes secreted by IECs has also been reported to contain considerable amounts of TGF-β [46], as also found in the present study (Table 2; Additional file 1: Figure S1). Likewise, the MHC II antigen-specific response in mice plasma-derived EVs has been reported [47]. MHC II could have similarly carried specific *E. falciformis* antigens to local APCs to initiate and/or remodel adaptive immune responses during parasite-induced inflammation [46]. Upregulation of MHC molecules in cEVs and sEVs in the present study (Table 3; Additional file 5, Additional file 6) suggests that the *E. falciformis* infection threshold transcended the host intestinal epithelium. As such, the host immune response to *E. falciformis* infection is not completely localized [48] and secreted EVs are important conveyors.

Plasma cell-derived Ig could be transported in vesicles to epithelial cell apices where the glycosylation of such Ig molecules could bind distinct intestinal microbes [49]. In addition, MHC molecules and ICAM-1 (CD54) on exosomes can bind to lymphocyte-associated antigen and T-cell immunoglobulin receptors [50] whose expression can modulate pro-inflammatory cytokines [3]. In the present study, several Ig molecules (Additional file 5, Additional file 6) were identified, but not IgA, possibly due to passive roles of humoral immunity against *Eimeria* infections [51]. Although *Eimeria* species are known to induce antibody production in the blood and mucosal secretions [21], our study shows that the majority of such molecules are likely secreted and transported via EVs. Additionally, non-specific immune factors, such as the complement system common in *Eimeria* infection [21], were contained in cEVs and sEVs and were differentially expressed (Additional file 5, Additional file 6).

Neutrophil granule and FAM3B proteins that attract eosinophil to enterocytes were significantly upregulated in cEVs (Additional file 6), suggesting a local innate immune response against *E. falciformis* [21]. Of note is the identification of CD5 antigen-like in sEVs at the time of oocyst shedding and host recovery (Table 1; Additional file 5), which is an indication of T cell-mediated systemic host immunity [21] against *E. falciformis* after the completion of intracellular development. Again, the regulatory variations in the pro-inflammatory cytokine receptors of IL-18 and macrophage colony-stimulating factor (mCSF) (Table 1) suggest that some host pro-inflammatory cytokines were constantly being switched on and off by *E. falciformis* or its derived antigens for the purpose of survival and life-cycle progression [30].

Similarly, STAT1 is important for interferon gamma (INF-γ), CD4^+^ and CD8^+^ T-lymphocyte production during *T. gondii* infection in mice [52]. As well, phosphorylation of STAT1 has been reported in acute mouse toxoplasmosis [53] and *Salmonella* infection with activation of caspase 8 and host cell death [54]. In the present study, upregulated STAT1 protein throughout *E. falciformis* infectious cycle in mice caecum-derived EVs was clearly a marker for mouse IEC death (Table 2). Functionally, STAT1 could mediate apoptosis and homeostasis during *E. falciformis* infection, suggesting a co-evolutionary protective and permissive measure by hosts and coccidian parasites. Also, isoforms of IEC-derived EV annexin could facilitate tissue repair [55] and function intermittently as the parasite destroys host cell during merozont development.

Intestinal barrier function is key to preventing pathogen-induced inflammation, but enteric pathogens can cause disruption of the epithelial tight junction and render it permeable to diverse cells and molecules [56]. The secretion of (tight) junction molecules in cEVs may also point to the destruction (and healing) process of the epithelial barrier during *E. falciformis* infection. Circulating EVs could participate in vascular homeostasis [47] and regulate various stimuli that could increase intracellular calcium levels for plasma membrane remodelling [50]. Calcium-activated chloride ion channel was found to be significantly upregulated in cEVs (Table 4). However, uptake of EVs by naïve or parasite-infected IECs possibly lead to Ca^2+^ influx [55], although the restriction of Ca^2+^ channels to sEVs suggests homeostatic imbalance during *E. falciformis* infection, and upregulated claudin (Additional file 5) may allow paracellular ion influx across the tight junction. Also, the presence of NADH dehydrogenase in cEVs alludes to the electron transport chain, as reported in *Eimeria acervuline* infection [57].

Microparticle-derived caspase 3 has been linked with various immune responses against protozoan parasites [58]. Caspase 3 and 9 have common effector cleavages and are implicated in apoptosis and pyroptosis [59] involving cytochrome* c* [60]. The present study showed upregulation of caspase 3 at the formation of the second merozont stage and at oocyst shedding/maturation, similar to the activation of caspase 3 reported in neutrophilic apoptosis during *Trichomonas vaginalis* infection [61]. The presence of cytochrome* c* and caspase 3 (Tables 2, 4) equally suggests a coordinated host response to eliminate *E. falciformis*-infected cells from the host caecal epithelium.

Additionally, the identification of vesicular gasdermin C protein points to membrane rupture by cell lysis and cell death [62], possibly due to neighbouring cells being alerted to potential dangers. While cytochrome* c* and apoptosis inducing factor (AIF) are crucial signaling molecules for apoptosis [63], NACHT, LRR and PYD domains-containing protein 6 (NLRP6) (Table 2) is the translational expression of NLRP6 inflammasomes associated with pyroptosis [64]. It was previously reported that *E. falciformis* sporozoite-derived EVs induced the upregulation of NLRP6 [30]. This indicates that *E. falciformis* and associated ligands could cause pyroptosis of IECs during infection. Also, the expression of EV programmed cell death proteins in sEVs and cEVs implies that *E. falciformis* infection involves host cell death, at least during the formation of merozonts (Tables 1, 2), and, specifically, the significant upregulation of calpain subunit in cEVs (Additional file 6) supports inflammasome-dependent IEC death [65] during *E. falciformis* development in the host. Although mucosal necrosis could characterize host cell death in chicken-infecting *Eimeria* species, it is likely that various, but distinct, forms of host cell death are mediated by *Eimeria* species depending on host genetics, intestinal niches and cascade of cellular signal received by infected host IECs, as depicted by the protein composition of secreted EVs.

sEVs contain more peptides with many unidentified proteins. Nonetheless, our protein search was limited to the mouse genome due to limitations in the number of peptides to which the spectrum can be matched as well as the intractable *E. falciformis* genome [66]. It is possible that these peptides (with yet-to-be identified proteins) were of parasite origin or uncharacterized EV-bound proteins induced by *E. falciformis*. Primarily, the proteomic data presented in the study are potential diagnostic molecules and disease indicators that can be curated for therapeutic purposes. Also, EVs can be isolated from the liver, thymus and spleen for comprehensive pathophysiological implication of EVs in murine and avian coccidiosis [22], as observed in experimental cryptosporidiosis [10]. Taken together, the present study presents EVs as conveyors of host regulatory proteins during *E. falciformis* infection and provides an overview of the parasite-host interaction via EVs across local and systemic levels in the mouse model of coccidiosis.

## Conclusion

Based on the biology and developmental transitions of *E. falciformis* in the host, sEVs and cEVs were processed and profiled. Host-derived sEV and cEV proteins varied greatly in function and quantity, reflecting the diverse biotic and physiological processes as *E. falciformis* changed forms in the host IECs. EV protein composition at the first and second merozont stages, at oocyst shedding and at the time of host recovery from the infection showed that local and systemic circulating EVs contained differentially regulated host proteins involved in innate and adaptive immune systems, ion transport, metabolism, cellular homeostasis and host cell death. sEV and cEV protein composition indicates that host responses to *E. falciformis* infection involve concerted humoral and cellular immune activities driven by secreted EVs. This study presents a quantitative proteomic assessment of EV proteins isolated from the host caecum and serum during *E. falciformis* infection across major infectious cycles. Some of the identified proteins are expected to form a critical basis for future work on the diagnostics and therapeutic targets in the control of coccidiosis.

### Supplementary Information

**Additional file 1: **Schematics, protein quantification and gene functions

**Additional file 2: **Protein peptide sequence

**Additional file 3: **sEV proteins

**Additional file 4: **cEV proteins

**Additional file 5: **sEV differentially expressed proteins

**Additional file 6: **cEV differentially expressed proteins

**Additional file 7: **GO terms for sEV proteins

**Additional file 8: **GO terms for cEV proteins

**Additional file 9: **KEGG terms for sEV proteins

**Additional file 10: **KEGG terms for cEV proteins

## Data Availability

All curated and analyzed data in this study are included in the published article and its additional files.
