# Supplementary material for: Quantitative proteomic analysis of local and systemic extracellular vesicles during Eimeria falciformis infectious cycle in the host
Source: Parasit Vectors. 2023 Sep 27;16:339. doi: 10.1186/s13071-023-05906-x (PMC10523797; doi:10.1186/s13071-023-05906-x)
Supplement: Supplementary file 1 — Additional file 1: Schematics, protein quantification and gene functions [file 13071_2023_5906_MOESM1_ESM.pdf]

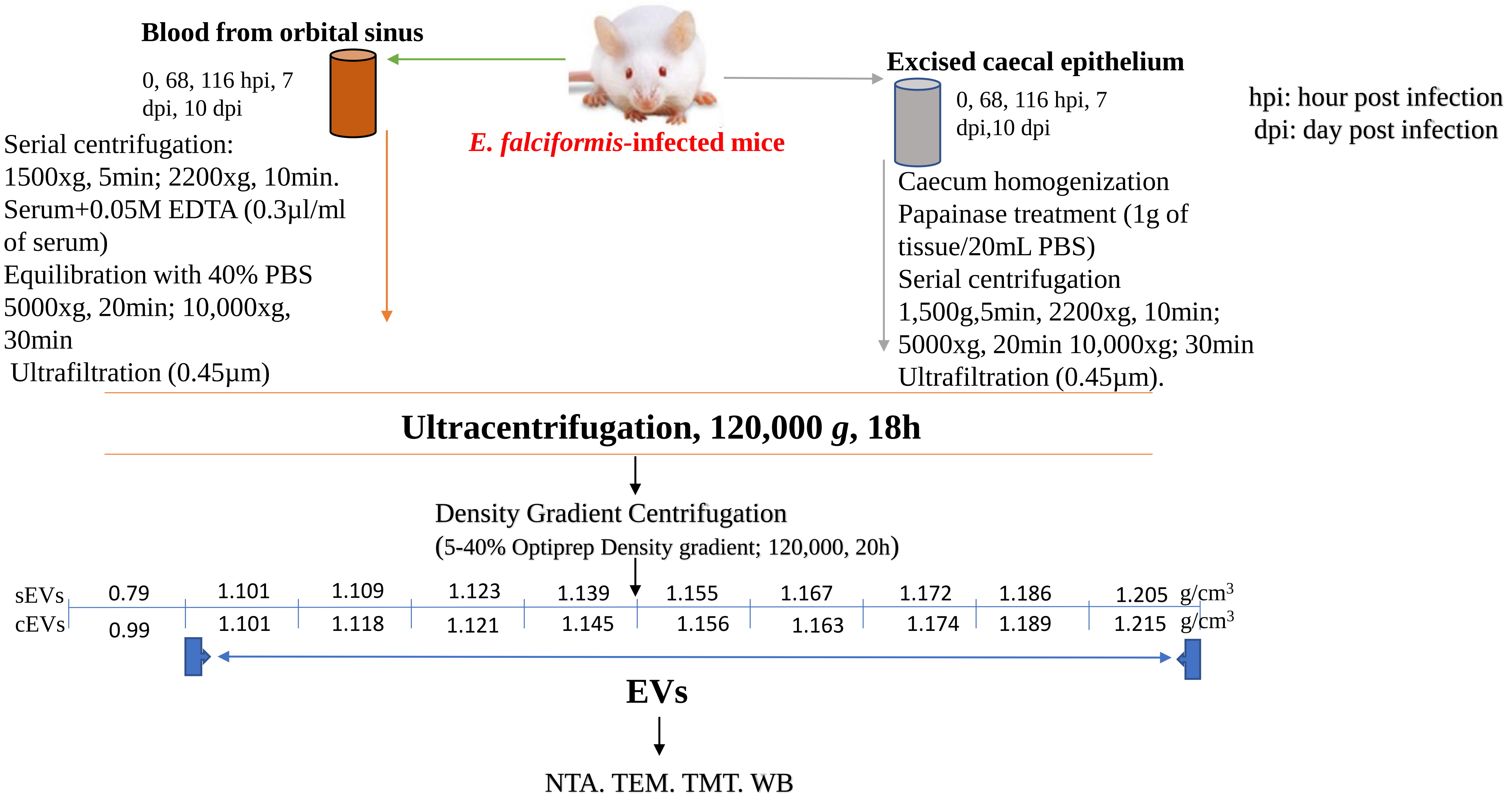

Fig. S1: Experimental workflow

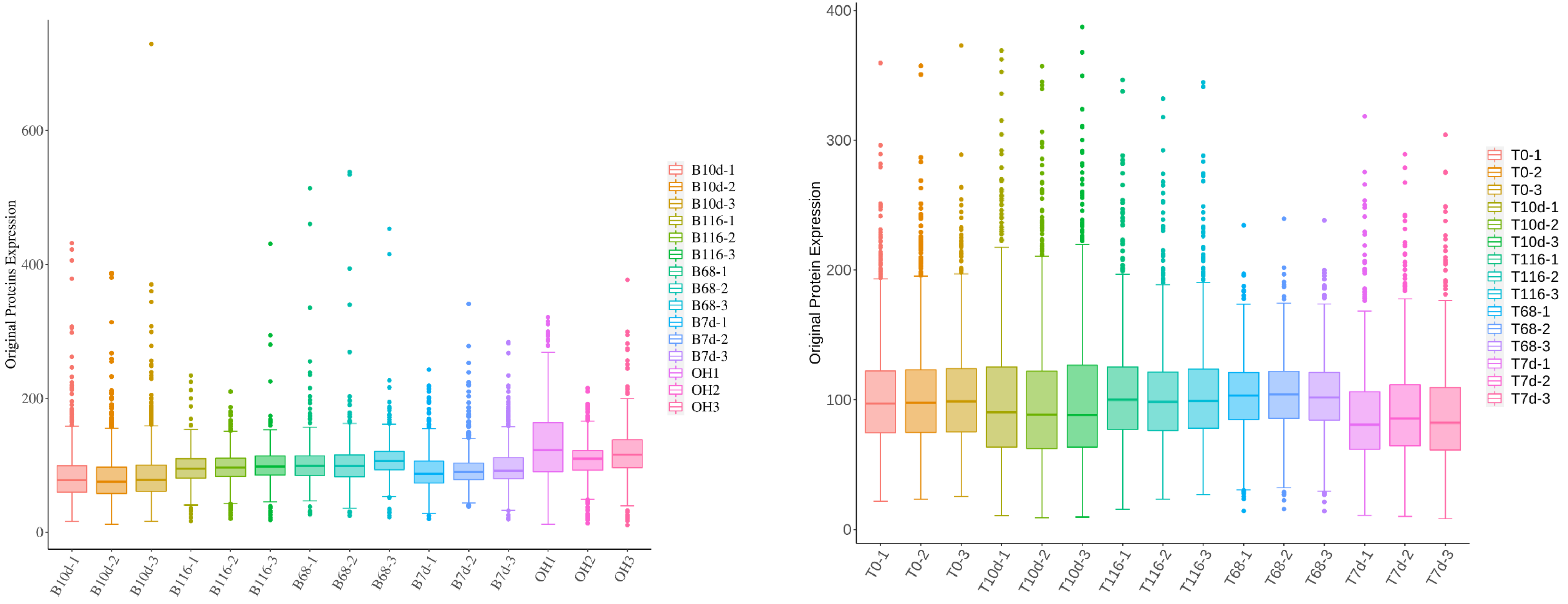

Fig. S2: Protein expression in blood serum (B) and caecal tissue (T) from *E. falciformis* infected mice

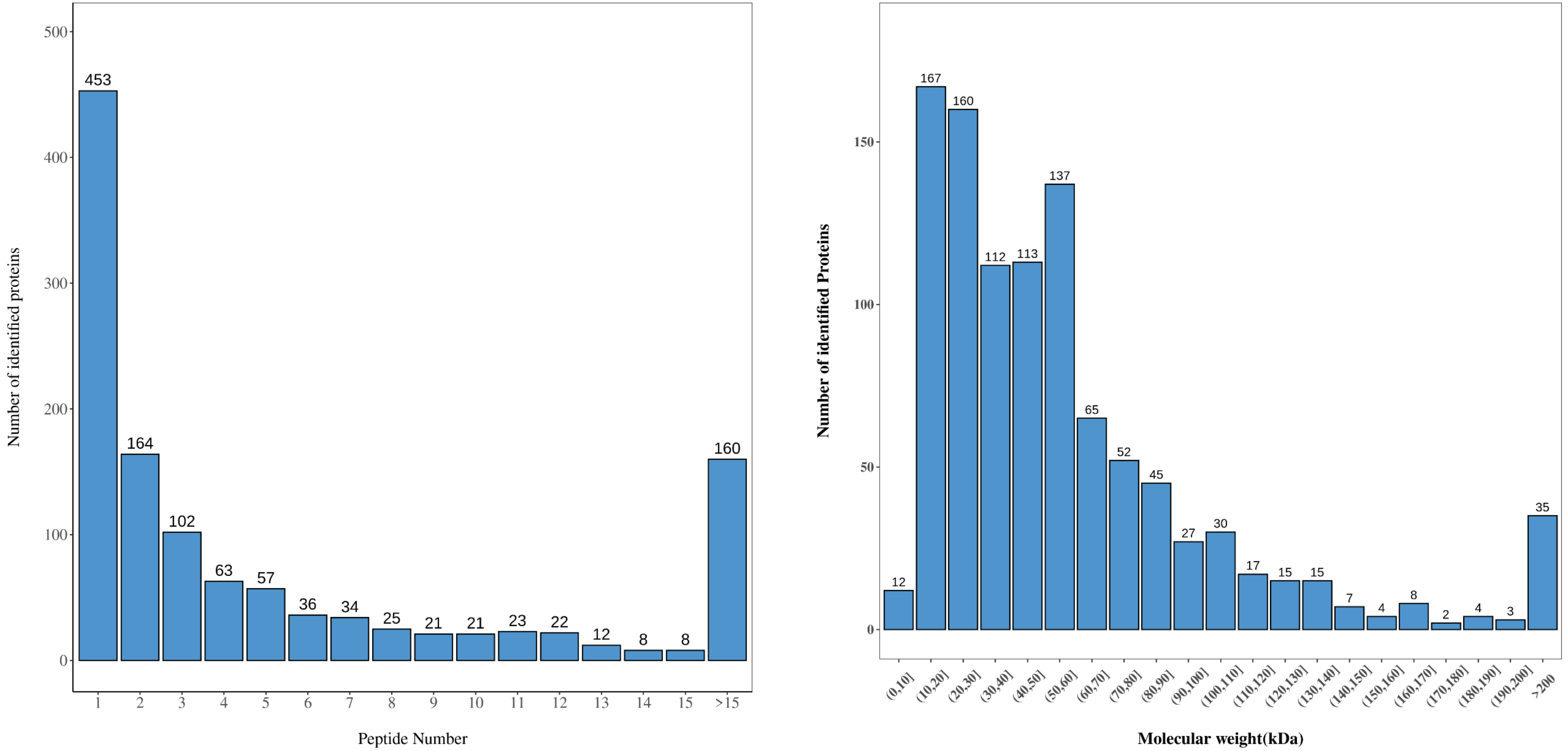

Fig. S3: sEV peptide and protein identification

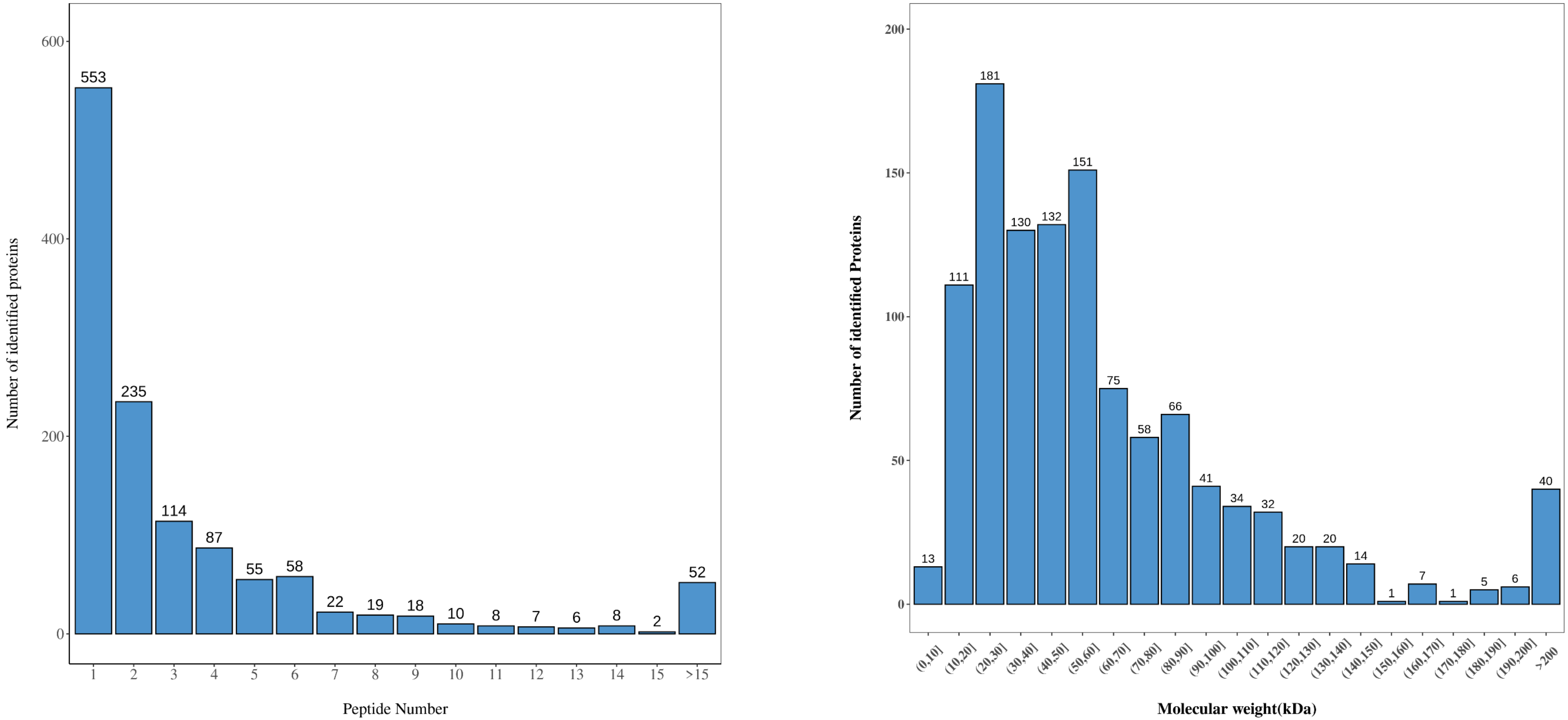

Fig. S4: cEV peptide and protein identification

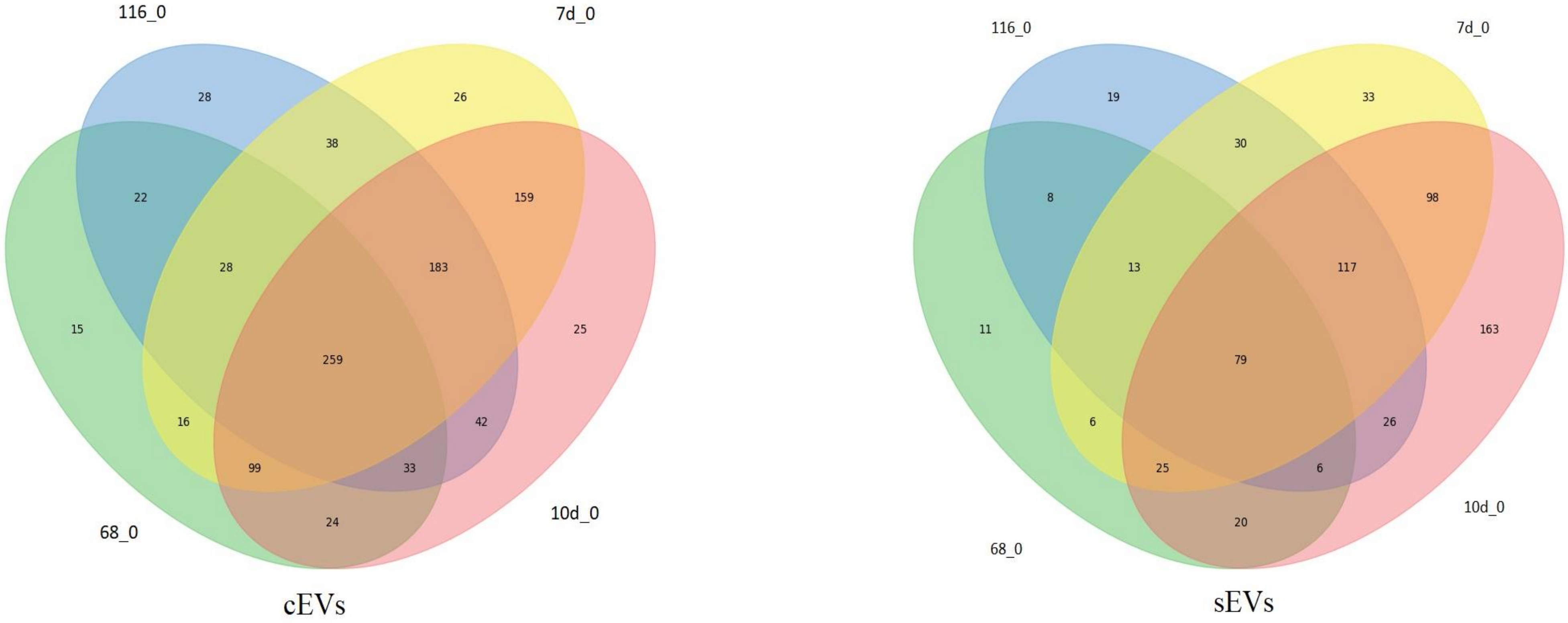

Fig. S5: Venn diagram for EV proteins across *E. falciformis* developmental stages in mice

35 proteins are commonly to serum- and caecum-derived Evs from *E. falciformis*-infected mice

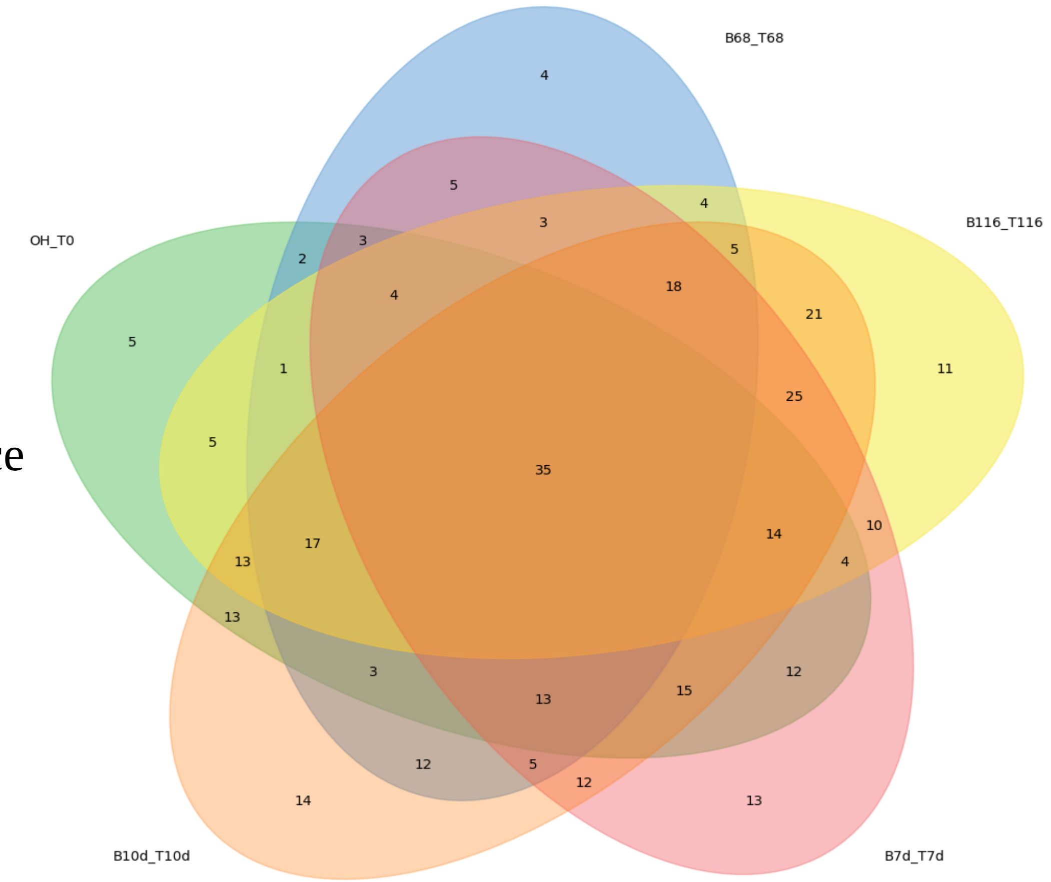

Fig. S6: Venn diagram for sEV and cEV proteins

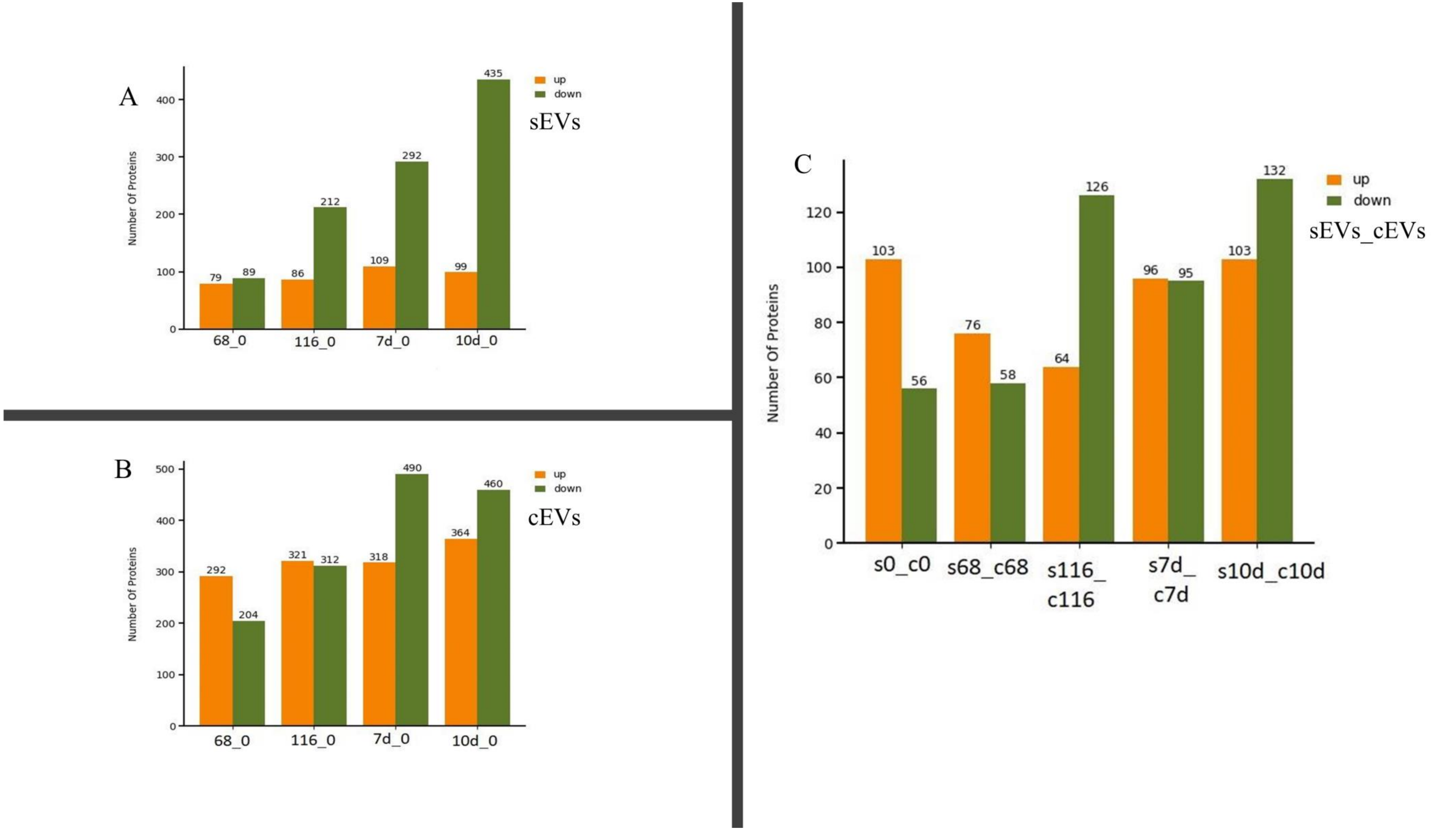

Fig. S7: Differential regulated proteins in the systemic and local EVs, A. up and down regulated sEV proteins B. up and down regulated cEV proteins C. differential regulated EV proteins in the serum and caecum of *E. falciformis*-infected mice

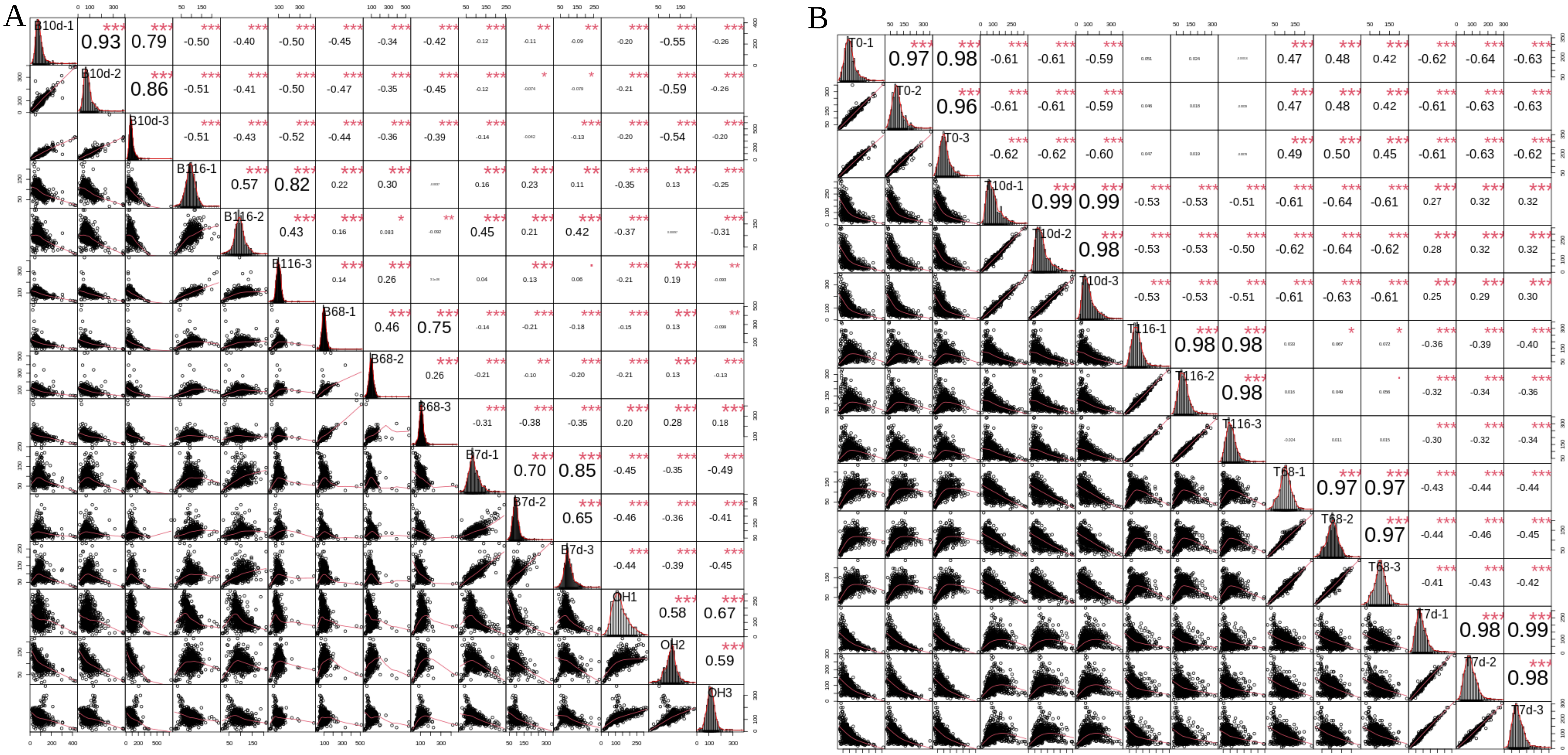

Fig. S8: Pearson correlation. A. sEV B. cEV proteins across *E. falciformis* developmental stages in mice

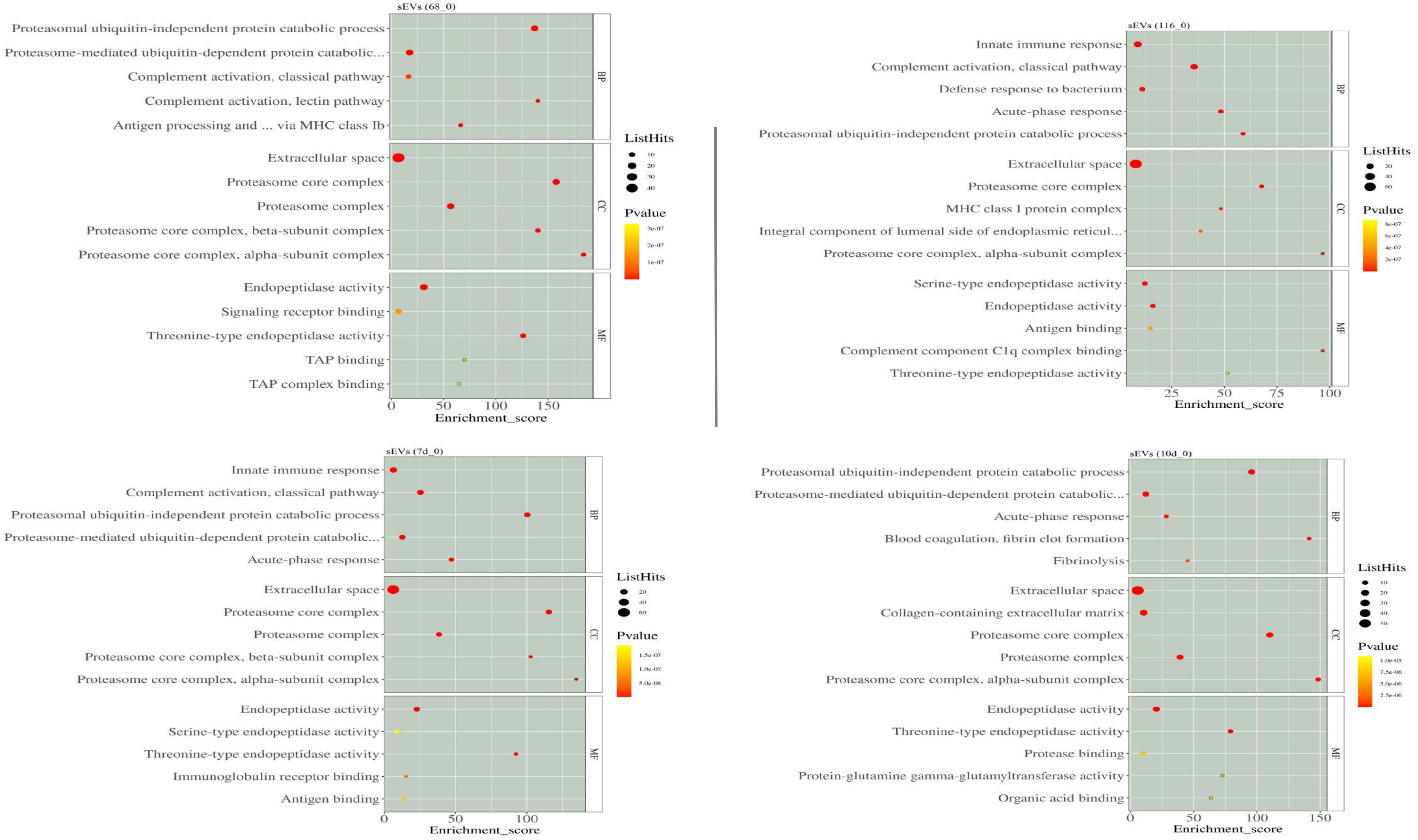

Fig. S9: GO terms for sEV proteins across *E. falciformis*-infected mice in comparison with 0h infection

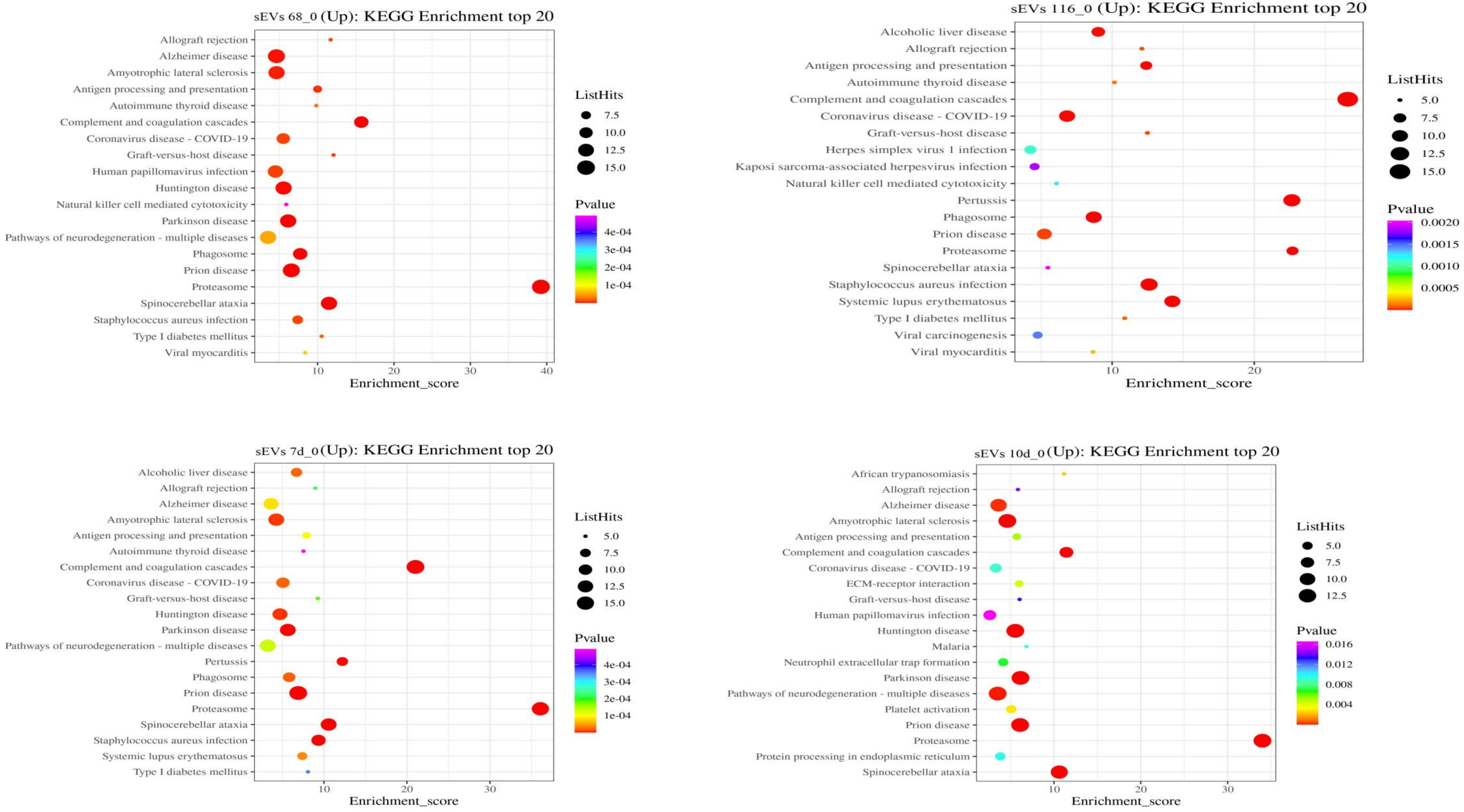

Fig. S10: KEGG terms for sEV proteins in *E. falciformis*-infected mice in comparison with 0h infection

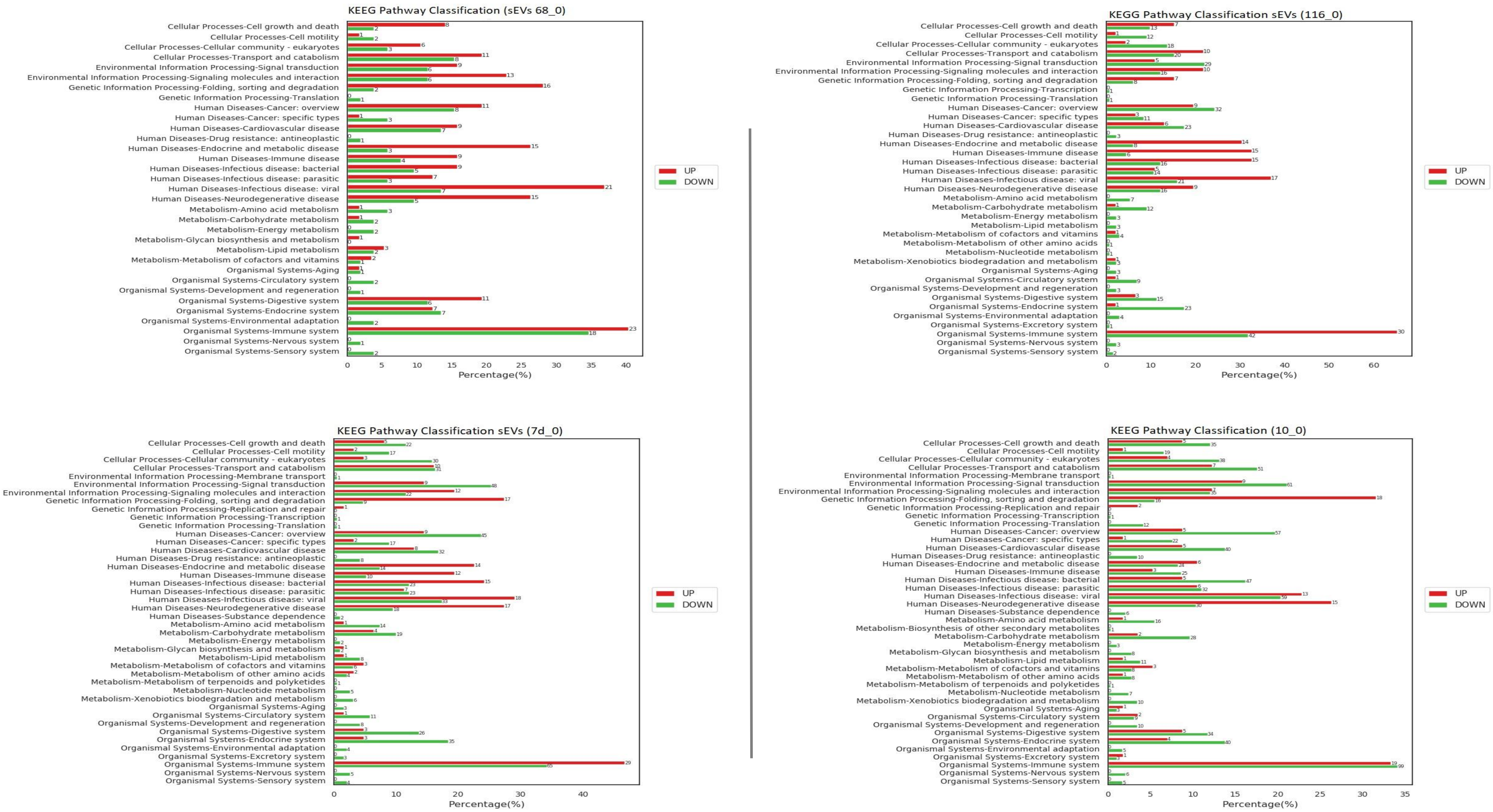

Fig. S11: KEGG enrichment pathway for sEV proteins across *E. falciformis*-infected mice in comparison with 0h infection

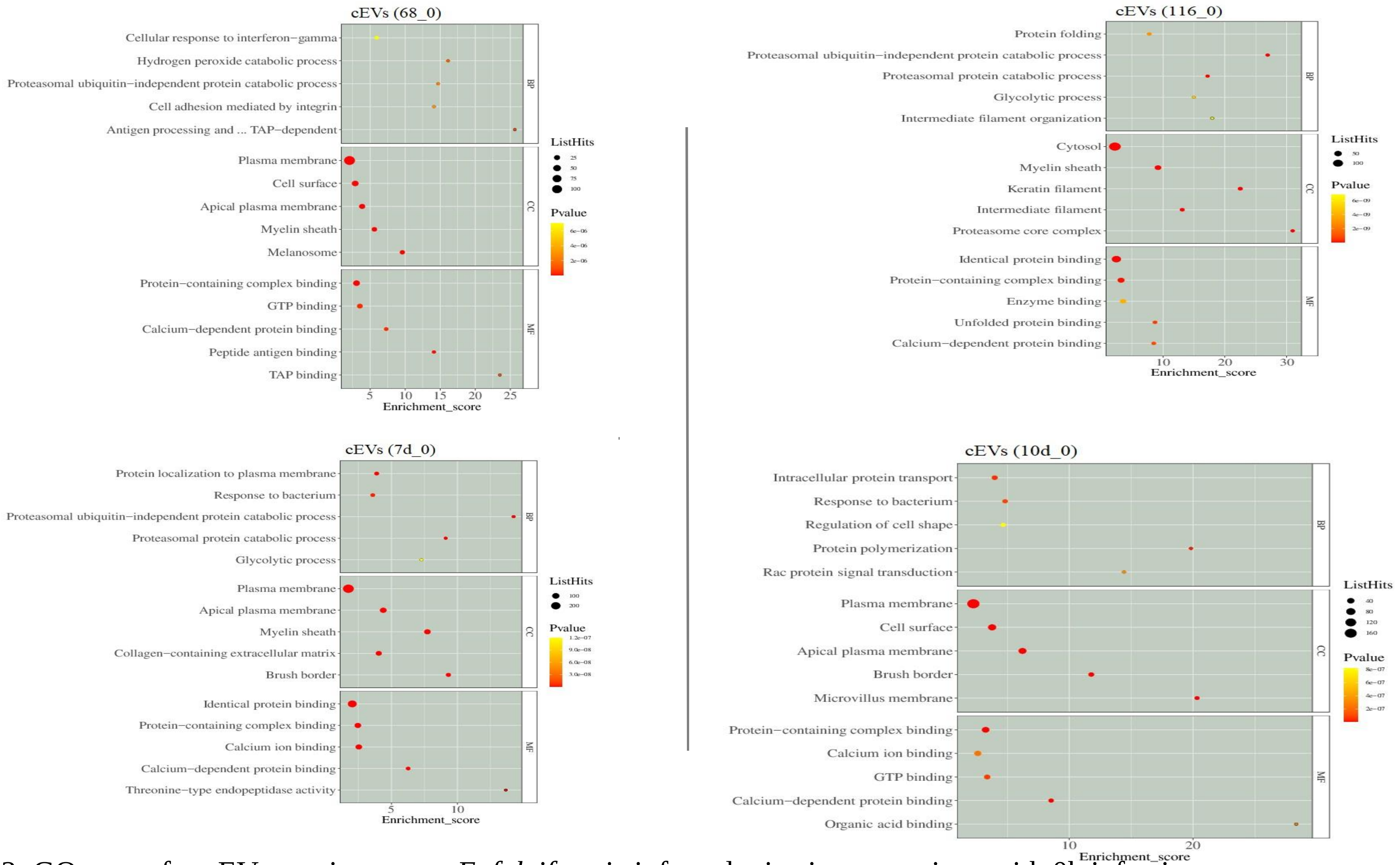

Fig. S12: GO terms for cEV proteins across *E. falciformis*-infected mice in comparison with 0h infection

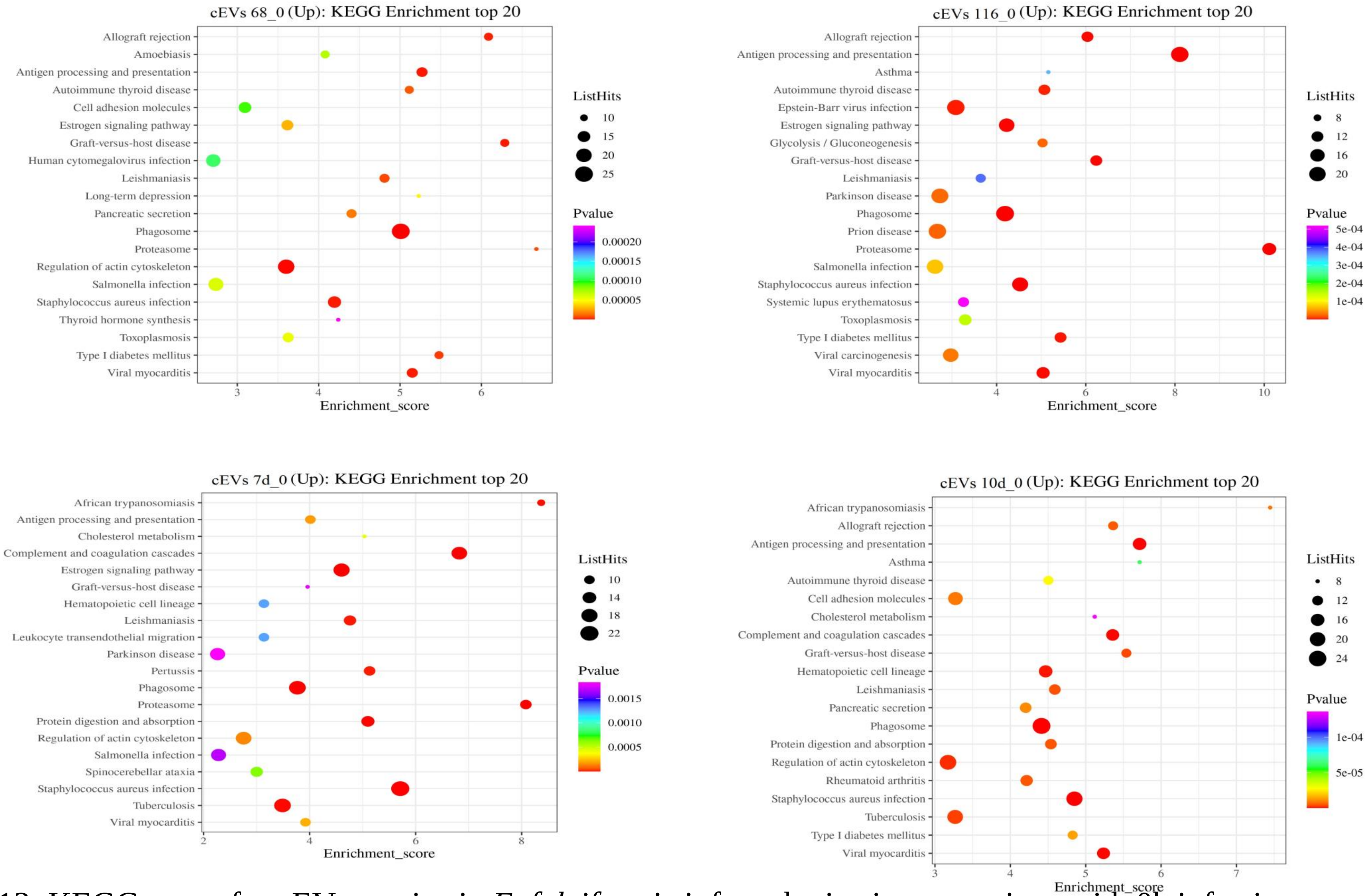

Fig. S13: KEGG terms for cEV proteins in *E. falciformis*-infected mice in comparison with 0h infection

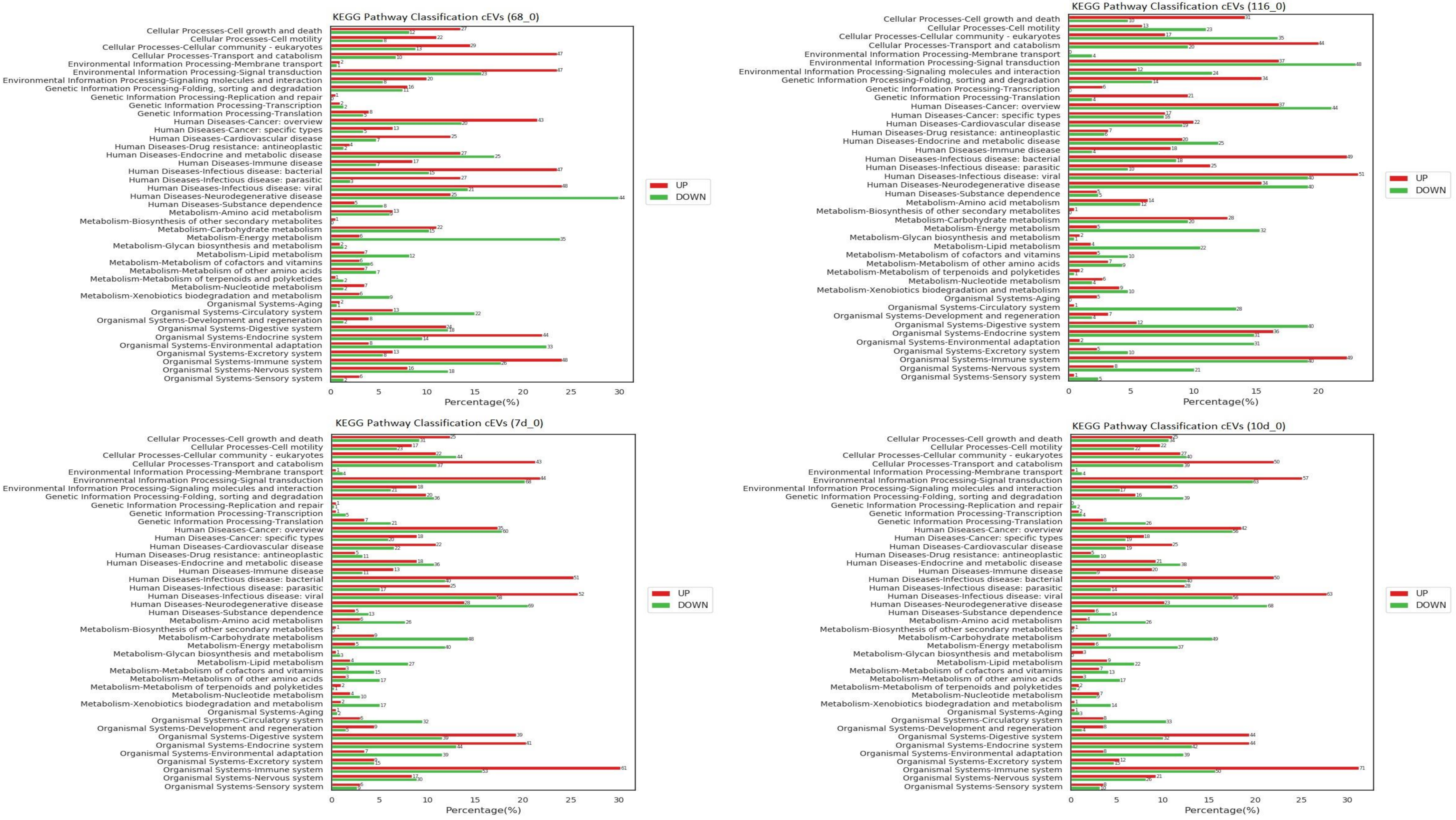

Fig. S14: KEGG enrichment pathway for cEV proteins across *E. falciformis*-infected mice in comparison with 0h infection
